# Supplementary material for: Characteristics of frequently attending children in hospital emergency departments: a systematic review
Source: BMJ Open. 2021 Oct 18;11(10):e051409. doi: 10.1136/bmjopen-2021-051409 (PMC8523960; doi:10.1136/bmjopen-2021-051409)
Supplement: Supplementary data [file bmjopen-2021-051409supp001.pdf]

Appendices

Appendix 1. Search terms

Search terms used in the Medline database.

1. exp Emergency Service, Hospital/
2. (Emergency adj3 (room* or department* or unit* or ward*)
3. (Trauma adj3 (room* or department* or unit* or ward*)
4. accident adj2 emergency
5. 1 or 2 or 3 or 4
6. adolescen* or child* or infant*
7. adolescent/ or exp child/ or exp infant/
8. p?ediatric*
9. baby or babies or teen* or girl* or boy*1
10. Youth*
11. young person*
12. young people
13. minor*
14. 6 or 7 or 8 or 9 or 10 or 11 or 13
15. frequen* adj3 (user* or attend* or usage* or admission* or visit*)
16. repeat* adj3 (user* or attend* or usage* or admission* or visit*)
17. heavy adj3 (user* or attend* or usage* or admission* or visit*)
18. Medical Overuse
19. over adj3 (user* or attend* or usage* or admission* or visit*)
20. super user*
21. superuser*
22. 15 or 16 or 17 or 18 or 19 or 20 or 21
23. 5 and 14 and 22
24. Limit 23 to English Language

Appendix 2. Study characteristics and main findings

Author	Year	Study Design	Country	Data source	Definition of Frequent Attendance (Attendances per year)	Age	Duration of Study	Number (proportion) of frequently attending children	Main findings
Yamamoto et al **[31]**	1995	Observational	USA	Kapiolani Medical Centre, Honolulu	>10	<21	4.6 years	72,049 (0.73%)	Frequent ED users had chronic disease conditions, neurological, gastrointestinal cardiac and endocrine conditions. Their Medical Insurance

									status included; private insurance, Medicaid/ state assistance, and no insurance
Laursen et al **[19]**	2006	Cohort	Denmark	National Patient Registry in Denmark	>5	<14	3 years	579,721 (0.43%)	Frequent ED users had more superficial injuries and more dislocations, sprains, and strains.
Gibson et al **[20]**	2010	Observational	Australia	Computerised Emergency Department Records, Perth Hospitals	>5	<15	6.5 years	229,883 (3%)	Factors associated with frequent attendances were; male, younger, self-referred, respiratory /infectious

									disorders, arrive by ambulance, higher admission rates & serious chronic illness
Markham et al **[33]**	2013	Case Control	Australia	Electronic Patient Records and Medical Records database, Monash Health	>8	<18	1 year	34,392 (0.38%)	Factors associated with frequent attendances were; female, arrive by ambulance, require admission for a mental health-related problem, discharge diagnosis related to oncology, neurology,

									respiratory, endocrinology and psychiatric complaints.
Alpern et al**[21]**	2014	Retrospective Cohort	USA	Paediatric Emergency Care Applied Research Network (PECARN)	>1	<17	1 year	695,188 (36%)	Asthma - most common ED diagnosis. Recurrent visits were associated with younger age, black or Hispanic race or ethnicity, and public health insurance
Cabey et al **[22]**	2014	Cross-sectional	USA	Patient Medical Records of	>5	<18	3 years	20,595 (28.5%)	Frequent PED use was associated with lack of primary care

				Carolinas Medical Center/Levin Children's Hospital					physician, nonprivate insurance, black race, Hispanic ethnicity, and residence in a poverty-associated zip code.
Neuman et al **[23]**	2014	Retrospective longitudinal	USA	Paediatric Health Information System, US	>1	<18	1 year	1,896,547 (75.19%)	Frequent user was had higher percentage of visits without medication administration, testing, or hospital admission & was usually <1 years old

Mueller et al **[24]**	2016	Retrospective Cohort	USA	Paediatric Health Information System (PHIS) Database, Colombia	>4	<19	1 year	17,943 (14.66%)	Frequent utilization was associated with age 1-19 years, Hispanic ethnicity, and urban residence. Few children with cancer received no medication, laboratory, or imaging during their ED visit.
Das et al **[30]**	2017	Retrospective Cohort	USA	Electronic Health Records (EHR) Systems,	>2	<18	1 year	2691(6.8%)	Frequent ED use was associated with co-morbidities, public insurance status, medication

				Weill Cornell Medical Center					history, and use of other healthcare resources. For publicly insured patients, the odds of frequent ED use were 3.1 times that of privately insured patients..
Christensen et al **[25]**	2017	Retrospective Cohort	USA	Medicaid	>4	<20	2 years	13,265 (8%)	Factors associated with frequent attendances were; chronic conditions, minority race, social determinants and young age.

Riva et al **[34]**	2018	Case-control	Italy	Administrative Healthcare databases of the Lombardy Region	>4	<18	1 year	1,640,713 (1.3%)	Trauma and respiratory tract infections were recognised as the most frequent diagnoses. Male gender, preschool age and place of residence were associated frequent ED use and frequent healthcare service use.
Supat et al **[26]**	2018	Retrospective Cohort	USA	California Office of Statewide	>6	<17	1 year	690,130 (2.3%)	Over 40% of frequent users visited 2 or more

				Health Planning and Development					hospitals. Frequent ED use were young age, publicly insured, and were admitted/transferred.
Blair et al **[32]**	2018	Observational	UK	District General Hospital Data, Brent and Harrow, London	>4	<5	1 years	10,169 (6.4%)	In the 10% sample, there were 304 attendances, and 69(23%) had an underlying chronic longstanding illness. This group were 2.4 times more likely to be admitted as in-patients.

Seguin et al **[27]**	2018	Retrospective Cohort	Canada	Systeme Informatique Urgence (SIURGE), electronic medical record database	>5	<18	1 year	52,088 (4.75%)	Lower socioeconomic status, Asthma, infectious ear, nose, and sinus disorders and other respiratory disorders were associated with a higher incidence of a recurrent visit.
Kuang et al **[35]**	2018	Nested Case Control	USA	California's Office of Statewide Health Planning	>2	<4	6 years	259,389 (1.45%)	For those with two or more ED visits Race/ethnicity, insurance status, and location of residence were also

				(OSHPD) and Development					significantly associated with the risk of child maltreatment.
Zook et al **[28]**	2018	Cross-sectional	USA	Emergency Department Medical Records in Minnesota and South Dakota	>4	<18	1 years	112,746 (4.68)	Native American patients had a high proportion of mental health diagnoses. Frequent ED visitors had greater odds of NA race, age younger than 1-year, public insurance, female sex, residence within less than 5 miles from

									the ED, and chronic disease.
Taylor et al **[29]**	2019	Cross Sectional	USA	Telephone survey, South Florida	>2	<17	1 year	1057 (16%)	Multiple ED visits were most strongly associated with access to care barriers attributed to language and culture, trouble finding a doctor, scheduling an appointment, and transportation access.
Hand et al **[37]**	2019	Cross Sectional	USA	Administrative billing data for	>10	12-17	6 years	5001 (5%)	Majority of high ED utilizers were male,

				ED visits, South Carolina (SC) Revenue and Fiscal Affairs (RFA) Office					with the exception of the ASD-only group which was predominantly female. The most common insurance type among high ED utilizers was either Medicare or Medicaid.
Vivier et al **[36]**	2019	Retrospective Cohort	USA	Statewide hospital network, Rhode Island	>4	<17	1 year	172,844 (5.2%)	Frequent ED users were often between the age of 0-1, Hispanic, Spanish-speaking, publicly insured and lived in

									areas with the highest neighbourhood risk. High ED utilizers were more associated with non-complex or complex chronic conditions and decreased distance to the ED.
Portley and North **[38]**	2020	Retrospective Cohort	USA	Children's Medical Center, The University of Texas Southwestern	>1	<18	1 year	6950 (10%)	Majority of frequent ED users were predominantly white English-speaking females who have private insurance

				Medical Center					and lived predominantly within the Dallas region of Texas, USA. Behavioural problems (34%) were the most common reason listed for the visit followed by suicidal ideation (24%) and suicide attempt (10%).
Leon et al **[39]**	2018	Retrospective Cohort	Canada	Children's Hospital of	>1	6-18	1 year	266 (26.3%)	Repeat ED visitors were commonly female who loved

				Eastern Ontario					within close proximity to the ED. Having a parent with a history for treatment of mental health (MH) was significantly associated with more frequent repeat visits to the ED. Furthermore, having more severe MH symptoms was associated with more frequent visits to the ED.

Appendix 3. Quality Assessment (Risk of Bias)

Quality Assessment of the included studies in the Systematic Review

Author	Study Design	Q1	Q2	Q3	Q4	Q5	Q6	Q7	Q8	Q9	Q10	Q11	Q12	Q13	Q14
Yamamoto et al [31]	Observational	Y	Y	Y	Y	N	Y	Y	Y	N/A	N	Y	N/A	N	N/A
Laursen et al [19]	Cohort	Y	Y	Y	Y	N	N	Y	N/A	N	N	Y	N	Y	Y
Gibson et al [20]	Observational	Y	Y	Y	Y	N	N	Y	N/A	Y	N	Y	N	N/A	Y
Alpern et al[21]	Retrospective Cohort	Y	Y	Y	Y	Y	Y	Y	N/A	Y	N	Y	N/A	Y	Y
Cabey et al [22]	Cross-sectional	Y	Y	Y	Y	N	N	Y	Y	Y	N	Y	N/A	Y	Y
Neuman et al [23]	Retrospective longitudinal	Y	Y	Y	Y	N	N	Y	Y	Y	N	Y	N/A	Y	Y
Mueller et al [24]	Retrospective Cohort	Y	Y	Y	Y	N	Y	Y	N	N/A	N	Y	N/A	N/A	Y
Das et al [30]	Retrospective Cohort	Y	Y	Y	Y	N	N	Y	N	N/A	N	Y	N/A	N/A	Y
Christensen et al [25]	Retrospective Cohort	Y	Y	Y	Y	N	Y	Y	N	N/A	N	Y	N/A	N	Y

Supat et al [26]	Retrospective cohort	Y	Y	Y	Y	N	Y	Y	N	Y	N	Y	N/A	N/A	Y
Blair et al [32]	Observational	Y	Y	Y	Y	N	Y	N	Y	N/A	N	Y	N/A	N/A	Y
Seguin et al [27]	Retrospective Cohort	Y	Y	Y	Y	N	N	Y	N	N/A	N	Y	N/A	Y	Y
Zook et al [28]	Cross-sectional, Cohort	Y	Y	Y	Y	N	N	Y	N	N/A	N	Y	N/A	N/A	Y
Leon et al [39]	Retrospective Cohort	Y	Y	Y	Y	N	Y	Y	N	N/A	N	Y	N/A	N/A	Y
Portley and North [38]	Retrospective Cohort	Y	Y	Y	Y	N	N	Y	N	N/A	N	Y	N/A	N/A	Y
Vivier et al [36]	Retrospective Cohort	Y	Y	Y	Y	N	Y	Y	N	N/A	N	Y	N/A	N/A	Y
Hand et al [37]	Cross Sectional	Y	Y	Y	Y	N	N	Y	N	N/A	N	Y	N/A	N/A	Y
Taylor et al [29]	Cross Sectional	Y	Y	Y	Y	N	Y	Y	N/A	N/A	N	Y	N/A	N	Y
Markham et al [33]	Retrospective case control	Y	Y	N	Y	Y	Y	Y	N	N	Y	N	Y		
Riva et al [34]	Case-control	Y	Y	N	Y	Y	N/A	Y	N	N	N	N	Y		

Kuang et al [35]	Nested Case Control	Y	Y	Y	N	Y	N/A	N/A	Y	Y	Y	Y	Y		

Legend:

Y Yes, N No, N/A (not applicable or not available)

Questions for the Tool for Quality Assessment of Observational Cohort and Cross-Sectional Studies [17]

1. Was the research question or objective in this paper clearly stated?
2. Was the study population clearly specified and defined?
3. Was the participation rate of eligible persons at least 50%?
4. Were all the subjects selected or recruited from the same or similar populations (including the same time period)? Were inclusion and exclusion criteria for being in the study prespecified and applied uniformly to all participants?
5. Was a sample size justification, power description, or variance and effect estimates provided?
6. For the analyses in this paper, were the exposure(s) of interest measured prior to the outcome(s) being measured?
7. Was the timeframe sufficient so that one could reasonably expect to see an association between exposure and outcome if it existed?
8. For exposures that can vary in amount or level, did the study examine different levels of the exposure as related to the outcome (e.g., categories of exposure, or exposure measured as continuous variable)?
9. Were the exposure measures (independent variables) clearly defined, valid, reliable, and implemented consistently across all study participants?
10. Was the exposure(s) assessed more than once over time?
11. Were the outcome measures (dependent variables) clearly defined, valid, reliable, and implemented consistently across all study participants?

12. Were the outcome assessors blinded to the exposure status of participants?
13. Was loss to follow-up after baseline 20% or less?
14. Were key potential confounding variables measured and adjusted statistically for their impact on the relationship between exposure(s) and outcome(s)?

Questions for the Tool for Quality Assessment of Case-Control Studies [17]

1. Was the research question or objective in this paper clearly stated and appropriate?
2. Was the study population clearly specified and defined?
3. Did the authors include a sample size justification?
4. Were controls selected or recruited from the same or similar population that gave rise to the cases (including the same timeframe)?
5. Were the definitions, inclusion and exclusion criteria, algorithms or processes used to identify or select cases and controls valid, reliable, and implemented consistently across all study participants?
6. Were the cases clearly defined and differentiated from controls?
7. If less than 100 percent of eligible cases and/or controls were selected for the study, were the cases and/or controls randomly selected from those eligible?
8. Was there use of concurrent controls?

9. Were the investigators able to confirm that the exposure/risk occurred prior to the development of the condition or event that defined a participant as a case?
10. Were the measures of exposure/risk clearly defined, valid, reliable, and implemented consistently (including the same time period) across all study participants?
11. Were the assessors of exposure/risk blinded to the case or control status of participants?
12. Were key potential confounding variables measured and adjusted statistically in the analyses? If matching was used, did the investigators account for matching during study analysis?
