# Supplementary material for: Characteristics of frequently attending children in hospital emergency departments: a systematic review
Source: BMJ Open. 2021 Oct 18;11(10):e051409. doi: 10.1136/bmjopen-2021-051409 (PMC8523960; doi:10.1136/bmjopen-2021-051409)
Supplement: Author's manuscript [file bmjopen-2021-051409.draft_revisions.pdf]

BMJ Open is committed to open peer review. As part of this commitment we make the peer review history of every article we publish publicly available.

When an article is published we post the peer reviewers' comments and the authors' responses online. We also post the versions of the paper that were used during peer review. These are the versions that the peer review comments apply to.

The versions of the paper that follow are the versions that were submitted during the peer review process. They are not the versions of record or the final published versions. They should not be cited or distributed as the published version of this manuscript.

BMJ Open is an open access journal and the full, final, typeset and author-corrected version of record of the manuscript is available on our site with no access controls, subscription charges or pay-per-view fees (http://bmjopen.bmj.com).

If you have any questions on BMJ Open's open peer review process please email info.bmjopen@bmj.com

Characteristics of frequently attending children in hospital emergency departments: A systematic review

Journal:	*BMJ Open*
Manuscript ID	bmjopen-2021-051409
Article Type:	Original research
Date Submitted by the Author:	18-Mar-2021
Complete List of Authors:	Greenfield, Geva; Imperial College London, School of Public Health<br>Okoli, Olivia; Imperial College London, School of Public Health<br>Quezada-Yamamoto, Harumi; Imperial College London, School of Public Health<br>Blair, Mitch; Imperial College London, Paediatrics<br>Saxena, Sonia; Imperial College London, School of Public Health<br>Majeed, Azeem; Imperial College London, Primary Care and Public Health<br>Hayhoe, Benedict; Imperial College London, School of Public Health
Keywords:	ACCIDENT & EMERGENCY MEDICINE, Paediatric intensive & critical care < INTENSIVE & CRITICAL CARE, Paediatric A&E and ambulatory care < PAEDIATRICS

SCHOLARONE™ Manuscripts

I, the Submitting Author has the right to grant and does grant on behalf of all authors of the Work (as defined in the below author licence), an exclusive licence and/or a non-exclusive licence for contributions from authors who are: i) UK Crown employees; ii) where BMJ has agreed a CC-BY licence shall apply, and/or iii) in accordance with the terms applicable for US Federal Government officers or employees acting as part of their official duties; on a worldwide, perpetual, irrevocable, royalty-free basis to BMJ Publishing Group Ltd ("BMJ") its licensees and where the relevant Journal is co-owned by BMJ to the co-owners of the Journal, to publish the Work in this journal and any other BMJ products and to exploit all rights, as set out in our licence.

The Submitting Author accepts and understands that any supply made under these terms is made by BMJ to the Submitting Author unless you are acting as an employee on behalf of your employer or a postgraduate student of an affiliated institution which is paying any applicable article publishing charge ("APC") for Open Access articles. Where the Submitting Author wishes to make the Work available on an Open Access basis (and intends to pay the relevant APC), the terms of reuse of such Open Access shall be governed by a Creative Commons licence – details of these licences and which Creative Commons licence will apply to this Work are set out in our licence referred to above.

Other than as permitted in any relevant BMJ Author's Self Archiving Policies, I confirm this Work has not been accepted for publication elsewhere, is not being considered for publication elsewhere and does not duplicate material already published. I confirm all authors consent to publication of this Work and authorise the granting of this licence.

For peer review only

Characteristics of frequently attending children in hospital emergency departments: A systematic review

Geva Greenfield PhD, Olivia Okoli MPH, Harumi Quezada Yamamoto MD, Mitch Blair MD, Sonia Saxena MD, Azeem Majeed MD, Benedict Hayhoe MD

Department of Primary Care and Public Health, School of Public Health, Imperial College London, UK

Affiliations: Department of Primary Care and Public Health, School of Public Health, Imperial College London, The Reynolds Building, St Dunstan's Road, London W6 8RP

Address correspondence to: Geva Greenfield, Department of Primary Care and Public Health, School of Public Health, Imperial College London, 314 The Reynolds Building, St Dunstan's Road, London W6 8RP, g.greenfield@ic.ac.uk

For peer review only

For peer review only - http://bmjopen.bmj.com/site/about/guidelines.xhtml

Abstract

Objective: To summarise the literature on frequent attendances to hospital emergency departments and describe sociodemographic and clinical characteristics of children who attend EDs frequently.

Setting: Hospital emergency departments.

Participants: Children <21 years, attending hospital emergency departments frequently.

Primary outcome measures: Outcomes measures were defined separately in each study, and were predominantly the number of ED attendances per year.

Results: We included 21 studies representing 6,513,627 children. Between 0.3% to 75% of all paediatric ED users were frequent users. Most studies defined 4 or more visits per year as a "frequent ED" usage. Children who were frequent ED users were more likely to be less than 5 years old. In the US, patients with public insurance were more likely to be frequent attenders. Frequent ED users more likely to be frequent users of primary care and have long-term conditions; the most common diagnoses were infections and gastroenteritis.

Conclusions: The review included a wide range of information across various health systems, however children who were frequent ED users have some universal characteristics in common. Policies to reduce frequent attendance might usefully focus on preschool children and supporting primary care in responding to primary-care oriented conditions.

For peer review only

Article summary

Strengths and limitations of this study

- This is the first systematic review we are aware of, summarising the literature on frequent attendances of children to hospital emergency departments.
- Young children are high users of EDs in general and a group with represents many frequent attendances.
- Children who were frequent ED users were more likely to be less than 5 years old and have long-term conditions
- Varied definitions for what constitute a frequent attendance introduces wide variations in the proportion of frequent attenders across the studies.
- Studies originating from different countries and healthcare systems makes comparisons between them challenging.

For peer review only

For peer review only - http://bmjopen.bmj.com/site/about/guidelines.xhtml

Funding/Support: This report is independent research supported by the National Institute for Health Research Applied Research Collaboration Northwest London. The views expressed in this publication are those of the authors and not necessarily those of the National Institute for Health Research or the Department of Health and Social Care. Grant No. NIHR200180. HQY was supported by the WHO Collaborating Centre for Public Health Education and Training at Imperial College London.

Competing interests statement. None of the authors have Conflict of Interest Disclosures.

Contributorship statement

Dr Greenfield, Ms Okoli and Dr Hayhoe were involved with conception and design, conducted the data analysis, and drafted the manuscript.

Ms Quezada Yamamoto was involved in conducting the literature searches, screening, data extraction and synthesis, and revised various versions of the manuscript.

Prof Blair, Prof Saxena and Prof Majeed were involved with acquisition of funding, conception and design, interpretation of the findings, provided clinical perspective, and revised various versions of the manuscript.

Data sharing statement

All data are available and could be shared upon request to the corresponding author.

Word count: 3,140 words, with one figure.

For peer review only

Introduction

Demand for health and emergency services is increasing rapidly driven by a worldwide epidemiological transition with increases in life expectancy, multi-morbidity, and burden from long-term conditions. For example, in England, the number of hospital emergency department (ED) attendances increased by 22% between 2008/09 to 2017/18 [1], while the growth in NHS England's funding having slowed significantly since 2010 [2], and approximately half of the NHS budget is currently spent on emergency and acute care, which is unsustainable in the long term [3]. These challenges have been intensified as health services are under increasing clinical and financial pressure amidst the COVID-19 pandemic.

Pressures on emergency health services are contributed to by a small group of individuals who attend EDs frequently, often referred to in the literature as 'Frequent attenders'. In some health systems, the total visits made by frequent users cost four times as much as for non-frequent users, representing a significant economic burden [4]. For example, in England, young children, along with the elderly, are the group with the highest number of frequent attendances [5]. Children under 5 years old, while being 5.7% of the population [6], account for 25.1% of all frequent attenders [5].

Approximately 15% of children ED attendances have been deemed inappropriate [7]. Nevertheless, the challenge with frequent ED visits is not only the over-utilisation of limited resources, but the potential harm that frequent exposure to ED can cause in terms of anxiety and distress [8], and an increased risk of infection [9,10].

Whilst frequent adult ED use has been reported in several reviews [11,12], there are currently two reviews of frequent children's ED attendances we are aware of. A review by Giannouchos et al. referred to American studies, and focused on mainstream excluded

For peer review only - http://bmjopen.bmj.com/site/about/guidelines.xhtml

specific clinical subgroups of children [13]. Poku el al. focused on effectiveness of interventions for non-urgent cases [14]. This study aimed at reviewing the literature of paediatric frequent attenders in hospital EDs, focusing on the volume of frequent attendance and the demographic and clinical features of frequent attenders, with a view to better understanding how services might respond efficiently to parent and carer demand for ED services.

Methods

The review was performed according to the recommendation in the Cochrane Handbook for Systematic Reviews of Interventions [15]. The PRISMA guideline for the reporting of systematic reviews and meta-analyses [16]. was also followed.

Eligibility Criteria

Studies published in English from all over the world were included to ensure that results are generalizable. No publication year limit was placed on the search, to ensure inclusion of as many relevant studies as possible.

Participants

Given that there is no universally accepted threshold for defining frequent ED use, we included studies which referred to children who frequently use EDs. Studies of paediatric patients aged 21 and below presenting to the ED were included. The 21 years threshold was chosen to capture as many relevant studies as possible.

Outcome(s)

For peer review only

For peer review only - http://bmjopen.bmj.com/site/about/guidelines.xhtml

Studies had to have a measurable, definitive outcome of frequent attendances, for example a number of attendances per child per year

Study Design

Studies providing quantitative data on frequently attending children in ED were included. RCTs, controlled before and after (CBA) studies, and interrupted time series (ITS) studies were included. Cohort and cross-sectional studies related to frequent attendances of children were also included. Qualitative designs and case reports were excluded. Although observational studies are conducted in uncontrolled conditions, we included such studies in the searches as they can provide further evidence of the outcomes of frequent ED attendances of children

Information Sources

Medline, Embase, Maternity and Infant Care, PsycINFO, HMIC, Global Health and Scopus were searched.

Search Strategy

The search strategy was developed with the support of a librarian specialist. Search terms such as “emergency department”, “accident adj2 emergency” were combined with search terms such as “adolescent* or child* or infant*” and “frequent admission/ visit” (Appendix 1). The search strategy was adapted for each electronic database and searches were run in June 2020.

Study Selection and Data Collection Process

The search results were transferred to Covidence (a systematic reviews management software) and further duplicates were removed. An initial screening of titles and abstracts was

For peer review only - http://bmjopen.bmj.com/site/about/guidelines.xhtml

followed by full-text screening of papers. The screening was done independently by OO, BH and GG. Discrepancies were resolved through discussion.

Data Extraction

The extracted information was collated into a structured format (Appendix 2). Additional information including sociodemographic information, reason(s) for visiting the ED, and relevant clinical information were extracted if were available.

Assessment of risk of bias in included studies

The risk of bias of included studies was assessed using the National Heart, lung and blood institute Quality Assessment Tool for Observational Cohort and Cross-sectional studies and Case Control studies [17]. It is an established and widely accepted Quality Assessment Tool. It was deemed appropriate because all included studies employed an observational study design. The criteria on the NIH Quality Assessment Tool are designed to help researchers focus on the key concepts for evaluating the internal validity of a study. They are not intended to create a list that could be tally up to arrive at a summary judgment of quality. The quality of the included studies was independently assessed by OO, BH and GG. Our responses to each of the 14 criteria are detailed in Table 3, followed by the criteria themselves. Discrepancies were resolved through discussion. Studies were not excluded based on risk of bias assessment.

Data Synthesis

Due to the heterogeneity in study designs and outcome measurements, a meta-analysis was deemed impractical. Instead a narrative synthesis approach was taken [18].

Patient and Public Involvement

Patients were not involved in the in the design of this study.

For peer review only - http://bmjopen.bmj.com/site/about/guidelines.xhtml

Results

Study Selection

Twenty-one studies representing 6,513,627 children met our inclusion criteria (Figure 1).

Study Characteristics

There were eighteen cohort studies [19–32] and three case control studies [33–35]. Fourteen were done in the USA, [21–26,28–31,35] two in Australia,[20,33] two in Canada,[27,36] one in the UK [32], one in Denmark [19] and one in Italy [34]. The study duration in most studies was one year. Frequently attending children percentage across all studies ranged from 0.3% to 75%. The data for all included studies were mainly extracted from ED registry, medical records and hospital admissions (Appendix 2).

Risk of bias within the included studies

Overall, the quality of all the included studies was high (Appendix 3). The main limitations related to risk of bias across the studies were sample size justification or power description, Measurement of exposure prior to the outcome being measured, and assessment of the exposure more than once over time.

Results of individual studies

Definitions and rates of frequent attendances

Most studies used more than four or five ED visits in one year [23–25,32,34,37], although the definition varied remarkably. The proportion of frequent attendances ranges from 0.3% to 75% of the overall number of subjects. An Australian study [20] referred to children attending extensively, more than 20 times in a year. In a review of studies from the United States, frequently attending adult and children represented approximately 4.5% to 8% of all

For peer review only - http://bmjopen.bmj.com/site/about/guidelines.xhtml

ED users and 21% to 28% of all ED visits [12], which is a narrow range than the range reported here.

Characteristics of frequently attending children

The identified characteristics amongst frequently attending children were classified into 3 groups: demographic factors; health service factors and clinical characteristics.

(1) Demographic factors

Age and sex. Twelve out of twenty-one studies reported that children under the age of 5 were more likely to be FA compared to their older children [20–24,26,27,31–35], Infants who were frequent attenders have a higher ED visit rate in preschool years (subsequent 2 years) than those who were not FA in infancy [22]. Five out of the thirteen studies reporting gender differences found that boys were more likely than girls to attend frequently [20,21,25,32,34], whereas four indicated that girls were more likely to attend frequently [26,30,33,35], and one study reported no significance difference between boys and girls [21].

Ethnic Group. Findings regarding the association between ethnic groups and frequent attendances were mixed and specific to different cultures [21–24,28,30–32]. In six studies [21–23,25,30,38], children from black (Non-Hispanic) groups were more likely to attend EDs than children from white background. Another study reported proportion of White, Non-Hispanic (24.7% vs 24.1%) and Hispanic (55.0% vs 53.3%) were approximately equal [34]. In another study, children from Hispanic groups was the most frequent paediatric ED users [24]. One UK study reported that children from south Asian or Asian British groups attended most frequently relatively to other groups [32].

Income. Children from households with lower incomes were more likely to attend EDs frequently compared to children from households with either median or higher incomes

For peer review only

[27,28,30,32]. Residence in a poverty was found in greater proportion in a frequent use group compared to a non-frequent use group [22]. One study [24] found an association between median household income and paediatric frequent ED usage, and another reported that actually children living in cities with an average/high income were 1.2 times more likely to have frequent non-urgent ED attendances relatively to children living in low income cities [34].

Geographical residence and proximity to an ED. Frequently attending children were more likely to live closer to the ED when compared with non-frequent ED users [23,28,31,36,39]. Four studies reported that living within an urban location [23,24,30,34] was associated with a higher likelihood of being a frequent ED user. However, another study reported that living in a rural setting was associated with frequent ED use [28].

(2) health service and health insurance factors

Health Insurance. In nine out of the thirteen studies reported on health insurance, frequently attending children were using public insurance [21,22,24–27,30–32], mainly Medicaid or State Assistance in the US . Patients with public insurance and uninsured patients had higher rates of frequent attendance compared with patients using private or commercial insurance [22,24,29,30,35].

Primary Care Provider. Among frequent ED users, the mean number of primary care visits per year was 6.2 visits [25]. Three studies indicated that frequent users of the ED were also more likely to be frequent users of primary care compared to non-frequent ED attenders [22–24]. One study found that frequently attending children who did not have a designated primary care physician were more likely to attend the ED more frequently compared to those who did have a primary care physician [22]. This was especially the case during the first

For peer review only - http://bmjopen.bmj.com/site/about/guidelines.xhtml

three years of life. Another study reported frequent ED users were more likely to live in a high PCP density area [23].

Access to healthcare barriers. Barriers to access to healthcare services, such as difficulty in finding physicians and booking appointments alongside transportation challenges, played a significant role in frequent ED use. Caregivers who experienced cultural or language barriers were 5.8 times more likely to take their child to the ED more than twice within the period of 12 months compared to children who did not have caregivers who experienced cultural or language barriers [29]. Transport challenges were also correlated with numerous ED visits [22].

(3) Clinical factors

Clinical diagnoses. Having chronic conditions were associated with high frequency ED use [21,23,25,26,28,29,31–33]. The most common diagnoses among frequently attending children were upper respiratory tract infections[20,21,23,25–27,34], accounting for approximately one third of all frequent attendances. Other common diagnoses were viral infections [20,23,27] , gastroenteritis [21,23,25] and mental health problems [36]. Frequently attending children were also more likely to have a discharge diagnosis related to oncology, neurology, respiratory, endocrinology and psychiatric complaints, compared with occasional attenders who were more likely to present with injury or trauma [21,33]. However, A Hawaiian study recognised that patients with chronic conditions often presented to the ED with problems which were not related to their chronic condition [31]. Allergies were less common among frequently attending children, they had a higher likelihood of acquiring prescriptions for asthma medication or medication related to other diseases [30].

For peer review only - http://bmjopen.bmj.com/site/about/guidelines.xhtml

Physical injuries and maltreatment. Frequently attending children presented with superficial injuries such as dislocations, sprains, strain injuries, contusion, open wounds and other trauma to the skin [19,23]. Most of these incidents occurred in children aged 6-11 years old and adolescents [34]. Young children with higher frequency of ED visits are at significantly increased risk of being victims of child maltreatment compared to occasional users [35].

Arrival, admission and discharge. Frequently attending children were more likely to arrive the ED by ambulance compared to occasional attenders [20,33]. They were more likely to have at least more than one ED visit leading to hospitalisation and/or transfer to another department, compared to non-frequent ED utilizers [21,24,33]. They were two times more likely to require admission for a mental health-related problem, compared to non-frequent ED utilizers [33]. They were 1.2 times more likely more likely to have received antibiotics and laboratory testing on discharge, compared to infrequent ED utilizers [24].

Discussion

Summary of main findings

The percentage of children who were frequent ED users ranged from 0.3% to 75%. Children who were frequent ED users were more likely to be aged less than 5 years, live in an urban location, near to an ED. Findings regarding sex, income and ethnicity were mixed. In the US, patients with public insurance were more likely frequent attendance compared with patients using private insurance. Frequent ED users more likely to be frequent users of primary care compared to non-frequent ED attenders. They tended to have long-term conditions and the most common diagnoses among frequently attending children were upper respiratory tract infections, viral infections and gastroenteritis.

For peer review only - http://bmjopen.bmj.com/site/about/guidelines.xhtml

Strengths and limitations

To our knowledge, this is the first systematic review to report on frequent ED attendance of children in a global perspective. Some challenges in the findings should be acknowledged. All the studies had observational design. The varied definitions for what constitute a frequent attendance, challenge the comparability of the studies, introducing wide variations in the proportion of frequent attenders between the studies. It is difficult to know whether the source of this variation is an outcome of differences in measurement methods, differences in populations, differences in healthcare systems structure or other sources. Likewise, the studies originated from different countries, cultures and healthcare systems, which makes comparisons between them challenging. Whereas most studies included all children <18 years, some studies focused on specific groups such as the <5 years old. Also, there was no blinding of the outcome assessor neither did majority of studies report whether there was a loss to follow-up. Finally, this systematic review restricted literature to only those that reported in English, which could potentially introduce selection bias.

Implications for future research

There is still little evidence on whether frequent ED users attend multiple ED sites or whether they are frequent users of other healthcare services. Linked data sets containing primary, secondary, hospital and social care data could be used for this purpose.

It would be useful to look at parent/carer characteristics as factors affecting frequently attending children. This is particularly important with younger children where the decision to attend the ED is made by the carer. This might highlight specific parent groups suspectable to frequent ED use.

From methodological perspective, using a uniform definition of will enable comparison across different studies. An alternative approach to the commonly used visit count approach

For peer review only - http://bmjopen.bmj.com/site/about/guidelines.xhtml

using a proportional threshold [40] (e.g., top 10% of all enlisted frequently attending children).

No data were found on any association between frequent ED attendances of children and risks of infection and other adverse health events following frequent ED visits. This is an important perspective to be looked at in future studies.

Implications for healthcare policy and practice

Creating tailored pathways to provide a response to the specific needs of frequently attending children are crucial. The characteristics of frequently attending children highlighted by this study can be used by healthcare providers to develop targeted interventions aimed at providing tailored response to the needs of frequent ED users either through primary care or other healthcare services. EDs could highlight paediatric patients attending EDs frequently to their GP practice so their GP could identify and seek to address unmet healthcare needs.

Proactive identification of children who frequently attend EDs at the primary care end (through flagging ED discharge summaries, or through EDs highlighting frequent attendance to the GP), and reviewing their situation to identify possible areas of concern or failure in support, could potentially reduce reliance on ED. Some ED triage services in England already take this approach, where a triage nurse may admit the patient to the ED or may redirect the patient to their GP, to an urgent care centre, a wellbeing service or social care service.

Frequent attendances might be a feature of publicly funded systems where services are widely available to patients at no charge. Indeed, US data show that patients with public insurance and the uninsured had higher rates of frequent attendance compared with those using private or commercial insurance.

For peer review only - http://bmjopen.bmj.com/site/about/guidelines.xhtml

But rather than trying to contain frequent attenders using caps and charges, it might be useful to understand why frequent users keep coming to the ED, and whether frequent attendances stem from pure clinical matters, psycho-social factors, lack of health literacy or self-care skills, for example among first time parents, or other factors.

Access to primary care is cited as a main driver in ED visits in children, particularly during out of hours. [3,41]" While access to primary care cannot be directly implied as a reason for frequent attenders, it might be a potential explanation for frequent attendances. If parents face challenges in accessing primary care, particularly during out-of-hours, they will understandably seek hospital care. Ensuring that primary care is accessible and effective in meeting the needs of children and their carers will therefore be essential in reducing unnecessary paediatric ED use. Children who use primary care benefit from health service accessibility, health prevention and early management of illnesses [42]. Improving access to primary care could hence reduce repeat ED use and improve overall long-term health.

Furthermore, as some clinical characteristics have been identified amongst frequently attending children, policy makers can implement national campaigns that will create awareness of what healthcare provider to visit with the appropriate associated symptoms.

Conclusion

The review included a wide range of information across various health systems, however children who were frequent ED users have some universal characteristics in common. We identified demographic factors, health service factors, and clinical characteristics relating to frequent ED attendances among children.

Policies to reduce frequent attendance might usefully focus on preschool children and supporting primary care in responding to primary-care oriented conditions. Further

For peer review only

investigation of the potential of tailored pathways to respond to the specific health needs of frequently attending children will be valuable in identification of effective interventions to reduce frequent attendance and improve children's long-term health. Specifically, policies to reduce frequent attendance might usefully focus on preschool children and supporting primary care in responding to primary-care oriented conditions.

For peer review only

For peer review only - http://bmjopen.bmj.com/site/about/guidelines.xhtml

References

1 NHS England. A&E Attendances and Emergency Admissions. https://www.england.nhs.uk/statistics/statistical-work-areas/ae-waiting-times-and-activity/ (accessed 29 Nov 2017).

2 King's Fund. How hospital activity in the NHS in England has changed over time. https://www.kingsfund.org.uk/publications/hospital-activity-funding-changes (accessed 25 Jun 2018).

3 Cecil E, Bottle A, Cowling TE, *et al.* Primary Care Access, Emergency Department Visits, and Unplanned Short Hospitalizations in the UK. *Pediatrics* 2016;**137**:e20151492. doi:10.1542/peds.2015-1492

4 Boh C, Li H, Finkelstein E, *et al.* Factors Contributing to Inappropriate Visits of Frequent Attenders and Their Economic Effects at an Emergency Department in Singapore. *Acad Emerg Med* 2015;**22**:1025–33. doi:10.1111/acem.12738

5 Greenfield G, Blair M, Aylin PP, *et al.* Frequent attendances at emergency departments in England. *Emerg Med J* 2020;:emermed-2018-208189. doi:10.1136/emermed-2018-208189

6 Principal projection - UK population in age groups - Office for National Statistics. https://www.ons.gov.uk/peoplepopulationandcommunity/populationandmigration/populationprojections/datasets/tablea21principalprojectionukpopulationinagegroups (accessed 16 Feb 2021).

7 McHale P, Wood S, Hughes K, *et al.* Who uses emergency departments inappropriately and when - a national cross-sectional study using a monitoring data system. *BMC Med* 2013;**11**:258. doi:10.1186/1741-7015-11-258

8 Nager AL, Mahrer NE, Gold JI. State Trait Anxiety in the Emergency Department: An Analysis of Anticipatory and Life Stressors. *Pediatr Emerg Care* 2010;**26**:897–901. doi:10.1097/PEC.0b013e3181fe90eb

9 Quach C, Moore D, Ducharme F, *et al.* Do pediatric emergency departments pose a risk of infection? *BMC Pediatr* 2011;**11**:2. doi:10.1186/1471-2431-11-2

10 Quach C, McArthur M, McGeer A, *et al.* Risk of infection following a visit to the emergency department: a cohort study. *Can Med Assoc J* 2012;**184**:E232–9. doi:10.1503/cmaj.110372

11 Soril LJJ, Leggett LE, Lorenzetti DL, *et al.* Reducing Frequent Visits to the Emergency Department: A Systematic Review of Interventions. *PLOS ONE* 2015;**10**:e0123660. doi:10.1371/journal.pone.0123660

12 LaCalle E, Rabin E. Frequent Users of Emergency Departments: The Myths, the Data, and the Policy Implications. *Ann Emerg Med* 2010;**56**:42–8. doi:10.1016/j.annemergmed.2010.01.032

For peer review only - http://bmjopen.bmj.com/site/about/guidelines.xhtml

13 Giannouchos TV, Kum H, Foster MJ, *et al.* Characteristics and predictors of adult frequent emergency department users in the United States: A systematic literature review. *J Eval Clin Pract* 2019;**25**:420–33. doi:10.1111/jep.13137

14 Poku BA, Hemingway P. Reducing repeat paediatric emergency department attendance for non-urgent care: a systematic review of the effectiveness of interventions. *Emerg Med J* 2019;:emermed-2018-207536. doi:10.1136/emermed-2018-207536

15 Pollock M, Fernandes R, Becker L, *et al.* Cochrane Handbook for Systematic Reviews of Interventions. In: *The Cochrane Collaboration*. 2016. www.handbook.cochrane.org (accessed 16 Aug 2019).

16 Shamseer L, Moher D, Clarke M, *et al.* Preferred reporting items for systematic review and meta-analysis protocols (PRISMA-P) 2015: elaboration and explanation. *BMJ* 2015;**349**:g7647–g7647. doi:10.1136/bmj.g7647

17 Study Quality Assessment Tools | National Heart, Lung, and Blood Institute (NHLBI). https://www.nhlbi.nih.gov/health-topics/study-quality-assessment-tools (accessed 5 Aug 2019).

18 Rodgers M, Sowden A, Petticrew M, *et al.* Testing Methodological Guidance on the Conduct of Narrative Synthesis in Systematic Reviews: Effectiveness of Interventions to Promote Smoke Alarm Ownership and Function. *Evaluation* 2009;**15**:49–73. doi:10.1177/1356389008097871

19 Laursen B. Injury patterns in children with frequent emergency department visits. *Emerg Med J EMJ* 2006;**23**:59–60.

20 Gibson NP, Jelinek GA, Jiwa M, *et al.* Paediatric frequent attenders at emergency departments: A linked-data population study: Paediatric frequent attenders at emergency departments. *J Paediatr Child Health* 2010;**46**:723–8. doi:10.1111/j.1440-1754.2010.01829.x

21 Alpern ER, Clark AE, Alessandrini EA, *et al.* Recurrent and High-frequency Use of the Emergency Department by Pediatric Patients. *Acad Emerg Med* 2014;**21**:365–73. doi:10.1111/acem.12347

22 Cabey WV, MacNeill E, White LN, *et al.* Frequent Pediatric Emergency Department Use in Infancy and Early Childhood: *Pediatr Emerg Care* 2014;**30**:710–7. doi:10.1097/PEC.0000000000000233

23 Neuman MI, Alpern ER, Hall M, *et al.* Characteristics of Recurrent Utilization in Pediatric Emergency Departments. *PEDIATRICS* 2014;**134**:e1025–31. doi:10.1542/peds.2014-1362

24 Mueller EL, Hall M, Carroll AE, *et al.* Frequent Emergency Department Utilizers Among Children with Cancer. *Pediatr Blood Cancer* 2016;**63**:859–64. doi:10.1002/pbc.25929

For peer review only - http://bmjopen.bmj.com/site/about/guidelines.xhtml

25 Christensen EW, Kharbanda AB, Veiden HV, *et al.* Predicting Frequent Emergency Department Use by Pediatric Medicaid Patients. *Popul Health Manag* 2017;**20**:208–15. doi:10.1089/pop.2016.0051

26 Supat B, Brennan JJ, Vilke GM, *et al.* Characterizing pediatric high frequency users of California emergency departments. *Am J Emerg Med* Published Online First: 2018. doi:10.1016/j.ajem.2018.12.015

27 Seguin J, Osmanlliu E, Zhang X, *et al.* Frequent users of the pediatric emergency department. *CJEM* 2018;**20**:401–8. doi:10.1017/cem.2017.15

28 Zook HG, Kharbanda AB, Puumala SE, *et al.* Emergency Department Utilization by Native American Children: *Pediatr Emerg Care* 2018;**34**:802–9. doi:10.1097/PEC.0000000000001289

29 Taylor T, Salyakina D. Health Care Access Barriers Bring Children to Emergency Rooms More Frequently: A Representative Survey. *Popul Health Manag* 2019;**22**:262–71. doi:10.1089/pop.2018.0089

30 Das LT, Abramson EL, Stone AE, *et al.* Predicting frequent emergency department visits among children with asthma using EHR data. *Pediatr Pulmonol* 2017;**52**:880–90. doi:10.1002/ppul.23735

31 Yamamoto LG, Zimmerman KR, Butts RJ, *et al.* Characteristics of frequent pediatric emergency departments users. *Pediatr Emerg Care* 1995;**11**:340–6. doi:0749.5161/95/1106.0340/$03.33/0

32 Blair M, Poots A, Lim V, *et al.* Pre-school children who are frequent attenders in Emergency Departments: an observational study of associated demographics and clinical characteristics. *Arch Dis Child* 2017;**103**:19–23. doi:https://dx.doi.org/10.1136/archdischild-2016-311952

33 Markham D, Graudins A. Characteristics of paediatric frequent presenters to an Australian emergency medicine network: Paediatric presenters to emergency. *J Paediatr Child Health* 2013;**49**:950–4. doi:10.1111/jpc.12288

34 Riva B, Clavenna A, Cartabia M, *et al.* Emergency department use by paediatric patients in Lombardy Region, Italy: a population study. *BMJ Paediatr Open* 2018;**2**:e000247. doi:10.1136/bmjpo-2017-000247

35 Kuang X, Aratani Y, Li G. Association between emergency department utilization and the risk of child maltreatment in young children. *Inj Epidemiol* 2018;**5**:46. doi:10.1186/s40621-018-0176-5

36 Leon SL, Polihronis C, Cloutier P, *et al.* Family Factors and Repeat Pediatric Emergency Department Visits for Mental Health: A Retrospective Cohort Study. *J Can Acad Child Adolesc Psychiatry* 2019;**28**:9–20.

For peer review only

For peer review only - http://bmjopen.bmj.com/site/about/guidelines.xhtml

37 Vivier PM, Rogers ML, Gjelsvik A, *et al.* Frequent Emergency Department Use by Children. *Pediatr Emerg Care* Published Online First: 11 July 2019. doi:10.1097/PEC.0000000000001859

38 Hand BN, Boan AD, Bradley CC, *et al.* Emergency department utilization and monetary charges in adolescents with autism spectrum disorder, intellectual disability, and a population comparison group. *Autism Res* 2019;**12**:1129–38. doi:10.1002/aur.2124

39 Portley R, North CS. Are There Differences in Pediatric Psychiatric Emergency Department Patients Related to Utilization Frequency? *Pediatr Emerg Care* 2020;**Publish Ahead of Print**. doi:10.1097/PEC.0000000000002010

40 Smits F, Brouwer H, ter Riet G. Persistent frequent attenders. *Br J Gen Pract* 2010;**60**:293.3-294. doi:10.3399/bjgp10X484002

41 Martin A, Martin C, Martin PB, *et al.* 'Inappropriate' attendance at an accident and emergency department by adults registered in local general practices: how is it related to their use of primary care? *J Health Serv Res Policy* 2002;**7**:160–5.

42 Starfield B, Shi L, Macinko J. Contribution of Primary Care to Health Systems and Health. *Milbank Q* 2005;**83**:457–502. doi:10.1111/j.1468-0009.2005.00409.x

For peer review only

Figures

Figure 1: PRISMA Flowchart

For peer review only

For peer review only - http://bmjopen.bmj.com/site/about/guidelines.xhtml

For peer review only

For peer review only - http://bmjopen.bmj.com/site/about/guidelines.xhtml

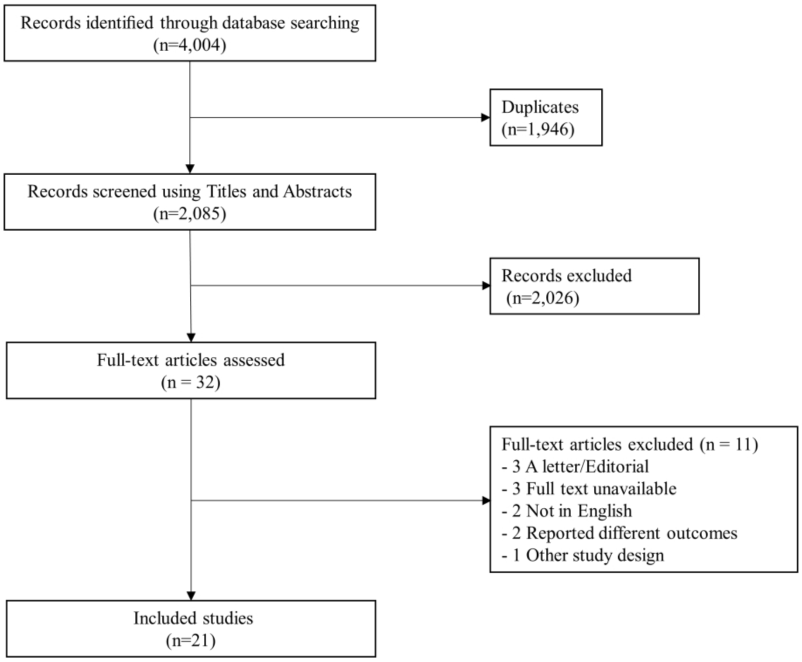

190x275mm (300 x 300 DPI)

Appendices

Appendix 1. Search terms

Search terms used in the Medline database.

1. exp Emergency Service, Hospital/
2. (Emergency adj3 (room* or department* or unit* or ward*)
3. (Trauma adj3 (room* or department* or unit* or ward*)
4. accident adj2 emergency
5. 1 or 2 or 3 or 4
6. adolescen* or child* or infant*
7. adolescent/ or exp child/ or exp infant/
8. p?ediatric*
9. baby or babies or teen* or girl* or boy*1
10. Youth*
11. young person*
12. young people
13. minor*
14. 6 or 7 or 8 or 9 or 10 or 11 or 13
15. frequen* adj3 (user* or attend* or usage* or admission* or visit*)
16. repeat* adj3 (user* or attend* or usage* or admission* or visit*)
17. heavy adj3 (user* or attend* or usage* or admission* or visit*)
18. Medical Overuse
19. over adj3 (user* or attend* or usage* or admission* or visit*)
20. super user*
21. superuser*
22. 15 or 16 or 17 or 18 or 19 or 20 or 21
23. 5 and 14 and 22
24. Limit 23 to English Language

For peer review only

Appendix 2. Study characteristics and main findings

Author	Year	Study Design	Country	Data source		Definition of Frequent Attendance (Attendances per year)	Age	Duration of Study	Number (proportion) of frequently attending children	Main findings
Yamamoto et al **[31]**	1995	Observational Study	USA	Kapiolani Medical Centre, Honolulu		>10	<21	4.6 years	72,049 (0.73%)	Frequent ED users conditions, neurolo cardiac and endocr Medical Insurance private insurance, I assistance, and no i
Laursen et al **[19]**	2006	Cohort Study	Denmark	National Patient Registry in Denmark		>5	<14	3 years	579,721 (0.43%)	Frequent ED users injuries and more c and strains.

Gibson et al **[20]**	2010	Observational Study	Australia	Computerised Emergency Department Records, Perth Hospitals		>5	<15	6.5 years	229,883 (3%)	Factors associated attendances were; r referred, respirator arrive by ambulanc rates & serious chr
Markham et al **[33]**	2013	Case Control study	Australia	Electronic Patient Records and Medical Records database, Monash Health		>8	<18	1 year	34,392 (0.38%)	Factors associated attendances were; f ambulance, require mental health-relat diagnosis related to respiratory, endocr psychiatric compla
Alpern et al**[21]**	2014	Retrospective Cohort Study	USA	Paediatric Emergency Care Applied		>1	<17	1 year	695,188 (36%)	Asthma - most com Recurrent visits we

For peer review only

				Research Network (PECARN)						younger age, black ethnicity, and publ
Cabey et al **[22]**	2014	Cross-sectional study	USA	Patient Medical Records of Carolinas Medical Center/Levin Children's Hospital		>5	<18	3 years	20,595 (28.5%)	Frequent PED use lack of primary car nonprivate insuran Hispanic ethnicity, poverty-associated
Neuman et al **[23]**	2014	Retrospective longitudinal study	USA	Paediatric Health Information System, US		>1	<18	1 year	1,896,547 (75.19%)	Frequent user was of visits without m administration, test admission & was u

For peer review only

Mueller et al **[24]**	2016	Retrospective Cohort Study	USA	Paediatric Health Information System (PHIS) Database, Colombia		>4	<19	1 year	17,943 (14.66%)	Frequent utilizatio age 1-19 years, His urban residence. Fe cancer received no laboratory, or imag visit.
Das et al **[30]**	2017	Retrospective Cohort Study	USA	Electronic Health Records (EHR) Systems, Weill Cornell Medical Center		>2	<18	1 year	2691(6.8%)	Frequent ED use w morbidities, public medication history healthcare resource insured patients, th use were 3.1 times insured patients..

Christensen et al **[25]**	2017	Retrospective Cohort Study	USA	Medicaid		>4	<20	2 years	13,265 (8%)	Factors associated attendances were; minority race, soci young age.
Riva et al **[34]**	2018	Case-control Study	Italy	Administrative Healthcare databases of the Lombardy Region		>4	<18	1 year	1,640,713 (1.3%)	Trauma and respira were recognised as diagnoses. Male ge and place of reside frequent ED use an service use.
Supat et al **[26]**	2018	Retrospective Cohort study	USA	California Office of Statewide Health Planning and Development		>6	<17	1 year	690,130 (2.3%)	Over 40% of frequ more hospitals. Fre young age, publicl admitted/transferre

Blair et al **[32]**	2018	Observational Study	UK	District General Hospital Data, Brent and Harrow, London		>4	<5	1 years	10,169 (6.4%)	In the 10% sample attendances, and 69 underlying chronic This group were 2. be admitted as in-p
Seguin et al **[27]**	2018	Retrospective Cohort Study	Canada	Systeme Informatique Urgence (SIURGE), electronic medical record database		>5	<18	1 year	52,088 (4.75%)	Lower socioeconon infectious ear, nose and other respirato associated with a h recurrent visit.
Kuang et al **[35]**	2018	Nested Case Control	USA	California's Office of Statewide		>2	<4	6 years	259,389 (1.45%)	For those with two Race/ethnicity, insu location of residen

				Health Planning (OSHPD) and Development						significantly associ child maltreatment
Zook et al **[28]**	2018	Cross-sectional Study	USA	Emergency Department Medical Records in Minnesota and South Dakota		>4	<18	1 years	112,746 (4.68)	Native American p proportion of ment Frequent ED visito NA race, age youn insurance, female s less than 5 miles fr chronic disease.
Taylor et al **[29]**	2019	Cross Sectional Study	USA	Telephone survey, South Florida		>2	<17	1 year	1057 (16%)	Multiple ED visits associated with acc attributed to langua trouble finding a do appointment, and t

Hand et al **[37]**	2019	Cross Sectional Study	USA	Administrative billing data for ED visits, South Carolina (SC) Revenue and Fiscal Affairs (RFA) Office		>10	12-17	6 years	5001 (5%)	Majority of high E with the exception group which was p The most common high ED utilizers w Medicaid.
Vivier et al **[36]**	2019	Retrospective Cohort Study	USA	Statewide hospital network, Rhode Island		>4	<17	1 year	172,844 (5.2%)	Frequent ED users the age of 0-1, Hisp speaking, publicly areas with the high risk. High ED utili associated with no chronic conditions distance to the ED.

Portley and North **[38]**	2020	Retrospective Cohort Study	USA	Children's Medical Center, The University of Texas Southwestern Medical Center		>1	<18	1 year	6950 (10%)	Majority of freque[illegible] predominantly whi[illegible] females who have [illegible] lived predominantl[illegible] region of Texas, U[illegible] problems (34%) w[illegible] reason listed for th[illegible] suicidal ideation (2[illegible] attempt (10%).
Leon et al **[39]**	2018	Retrospective Cohort Study	Canada	Children's Hospital of Eastern Ontario		>1	6-18	1 year	266 (26.3%)	Repeat ED visitors [illegible] female who loved [illegible] to the ED. Having [illegible] for treatment of me[illegible] significantly associ[illegible] frequent repeat visi[illegible] Furthermore, havin[illegible]

For peer review

										symptoms was ass[illegible] frequent visits to th[illegible]

For peer review only

Appendix 3. Quality Assessment (Risk of Bias)

Quality Assessment of the included studies in the Systematic Review

Author	Study Design	Q1	Q2	Q3	Q4	Q5	Q6	Q7	Q8	Q9	Q10	Q11	Q12	Q13	Q14
Yamamoto et al [31]	Observational Study	Y	Y	Y	Y	N	Y	Y	Y	N/A	N	Y	N/A	N	N/A
Laursen et al [19]	Cohort Study	Y	Y	Y	Y	N	N	Y	N/A	N	N	Y	N	Y	Y
Gibson et al [20]	Observational Study	Y	Y	Y	Y	N	N	Y	N/A	Y	N	Y	N	N/A	Y
Alpern et al[21]	Retrospective Cohort Study	Y	Y	Y	Y	Y	Y	Y	N/A	Y	N	Y	N/A	Y	Y
Cabey et al [22]	Cross-sectional study	Y	Y	Y	Y	N	N	Y	Y	Y	N	Y	N/A	Y	Y
Neuman et al [23]	Retrospective longitudinal study	Y	Y	Y	Y	N	N	Y	Y	Y	N	Y	N/A	Y	Y
Mueller et al [24]	Retrospective Cohort Study	Y	Y	Y	Y	N	Y	Y	N	N/A	N	Y	N/A	N/A	Y
Das et al [30]	Retrospective Cohort Study	Y	Y	Y	Y	N	N	Y	N	N/A	N	Y	N/A	N/A	Y
Christensen et al [25]	Retrospective Cohort Study	Y	Y	Y	Y	N	Y	Y	N	N/A	N	Y	N/A	N	Y

For peer review only - http://bmjopen.bmj.com/site/about/guidelines.xhtml

Supat et al [26]	Retrospective cohort study	Y	Y	Y	Y	N	Y	Y	N	Y	N	Y	N/A	N/A	Y
Blair et al [32]	Observational Study	Y	Y	Y	Y	N	Y	N	Y	N/A	N	Y	N/A	N/A	Y
Seguin et al [27]	Retrospective Cohort Study	Y	Y	Y	Y	N	N	Y	N	N/A	N	Y	N/A	Y	Y
Zook et al [28]	Cross-sectional, Cohort Study	Y	Y	Y	Y	N	N	Y	N	N/A	N	Y	N/A	N/A	Y
Leon et al [39]	Retrospective Cohort Study	Y	Y	Y	Y	N	Y	Y	N	N/A	N	Y	N/A	N/A	Y
Portley and North [38]	Retrospective Cohort Study	Y	Y	Y	Y	N	N	Y	N	N/A	N	Y	N/A	N/A	Y
Vivier et al [36]	Retrospective Cohort Study	Y	Y	Y	Y	N	Y	Y	N	N/A	N	Y	N/A	N/A	Y
Hand et al [37]	Cross Sectional Study	Y	Y	Y	Y	N	N	Y	N	N/A	N	Y	N/A	N/A	Y
Taylor et al [29]	Cross Sectional Study	Y	Y	Y	Y	N	Y	Y	N/A	N/A	N	Y	N/A	N	Y
Markham et al [33]	Retrospective case control study	Y	Y	N	Y	Y	Y	Y	N	N	Y	N	Y		
Riva et al [34]	Case-control Study	Y	Y	N	Y	Y	N/A	Y	N	N	N	N	Y		

For peer review only

Kuang et al [35]	Nested Case Control	Y	Y	Y	N	Y	N/A	N/A	Y	Y	Y	Y	Y		

Legend:

Y Yes, N No, N/A (not applicable or not available)

For peer review only

Questions for the Tool for Quality Assessment of Observational Cohort and Cross-Sectional Studies [17]

1. Was the research question or objective in this paper clearly stated?
2. Was the study population clearly specified and defined?
3. Was the participation rate of eligible persons at least 50%?
4. Were all the subjects selected or recruited from the same or similar populations (including the same time period)? Were inclusion and exclusion criteria for being in the study prespecified and applied uniformly to all participants?
5. Was a sample size justification, power description, or variance and effect estimates provided?
6. For the analyses in this paper, were the exposure(s) of interest measured prior to the outcome(s) being measured?
7. Was the timeframe sufficient so that one could reasonably expect to see an association between exposure and outcome if it existed?
8. For exposures that can vary in amount or level, did the study examine different levels of the exposure as related to the outcome (e.g., categories of exposure, or exposure measured as continuous variable)?
9. Were the exposure measures (independent variables) clearly defined, valid, reliable, and implemented consistently across all study participants?
10. Was the exposure(s) assessed more than once over time?
11. Were the outcome measures (dependent variables) clearly defined, valid, reliable, and implemented consistently across all study participants?

For peer review only

12. Were the outcome assessors blinded to the exposure status of participants?
13. Was loss to follow-up after baseline 20% or less?
14. Were key potential confounding variables measured and adjusted statistically for their impact on the relationship between exposure(s) and outcome(s)?

Questions for the Tool for Quality Assessment of Case-Control Studies [17]

1. Was the research question or objective in this paper clearly stated and appropriate?
2. Was the study population clearly specified and defined?
3. Did the authors include a sample size justification?
4. Were controls selected or recruited from the same or similar population that gave rise to the cases (including the same timeframe)?
5. Were the definitions, inclusion and exclusion criteria, algorithms or processes used to identify or select cases and controls valid, reliable, and implemented consistently across all study participants?
6. Were the cases clearly defined and differentiated from controls?
7. If less than 100 percent of eligible cases and/or controls were selected for the study, were the cases and/or controls randomly selected from those eligible?
8. Was there use of concurrent controls?

For peer review only

9. Were the investigators able to confirm that the exposure/risk occurred prior to the development of the condition or event that defined a participant as a case?
10. Were the measures of exposure/risk clearly defined, valid, reliable, and implemented consistently (including the same time period) across all study participants?
11. Were the assessors of exposure/risk blinded to the case or control status of participants?
12. Were key potential confounding variables measured and adjusted statistically in the analyses? If matching was used, did the investigators account for matching during study analysis?

For peer review only

BMJ Open

Characteristics of frequently attending children in hospital emergency departments: A systematic review

Journal:	*BMJ Open*
Manuscript ID	bmjopen-2021-051409.R1
Article Type:	Original research
Date Submitted by the Author:	18-Jul-2021
Complete List of Authors:	Greenfield, Geva; Imperial College London, School of Public Health<br>Okoli, Olivia; Imperial College London, School of Public Health<br>Quezada-Yamamoto, Harumi; Imperial College London, School of Public Health<br>Blair, Mitch; Imperial College London, Paediatrics<br>Saxena, Sonia; Imperial College London, School of Public Health<br>Majeed, Azeem; Imperial College London, Primary Care and Public Health<br>Hayhoe, Benedict; Imperial College London, School of Public Health
**Primary Subject Heading**:	Emergency medicine
Secondary Subject Heading:	Paediatrics
Keywords:	ACCIDENT & EMERGENCY MEDICINE, Paediatric intensive & critical care < INTENSIVE & CRITICAL CARE, Paediatric A&E and ambulatory care < PAEDIATRICS

SCHOLARONE™ Manuscripts

I, the Submitting Author has the right to grant and does grant on behalf of all authors of the Work (as defined in the below author licence), an exclusive licence and/or a non-exclusive licence for contributions from authors who are: i) UK Crown employees; ii) where BMJ has agreed a CC-BY licence shall apply, and/or iii) in accordance with the terms applicable for US Federal Government officers or employees acting as part of their official duties; on a worldwide, perpetual, irrevocable, royalty-free basis to BMJ Publishing Group Ltd ("BMJ") its licensees and where the relevant Journal is co-owned by BMJ to the co-owners of the Journal, to publish the Work in this journal and any other BMJ products and to exploit all rights, as set out in our licence.

The Submitting Author accepts and understands that any supply made under these terms is made by BMJ to the Submitting Author unless you are acting as an employee on behalf of your employer or a postgraduate student of an affiliated institution which is paying any applicable article publishing charge ("APC") for Open Access articles. Where the Submitting Author wishes to make the Work available on an Open Access basis (and intends to pay the relevant APC), the terms of reuse of such Open Access shall be governed by a Creative Commons licence – details of these licences and which Creative Commons licence will apply to this Work are set out in our licence referred to above.

Other than as permitted in any relevant BMJ Author's Self Archiving Policies, I confirm this Work has not been accepted for publication elsewhere, is not being considered for publication elsewhere and does not duplicate material already published. I confirm all authors consent to publication of this Work and authorise the granting of this licence.

For peer review only

Characteristics of frequently attending children in hospital emergency departments: A systematic review

Geva Greenfield PhD, Olivia Okoli MPH, Harumi Quezada Yamamoto MD, Mitch Blair MD, Sonia Saxena MD, Azeem Majeed MD, Benedict Hayhoe MD

Department of Primary Care and Public Health, School of Public Health, Imperial College London, UK

Affiliations: Department of Primary Care and Public Health, School of Public Health, Imperial College London, The Reynolds Building, St Dunstan's Road, London W6 8RP

Address correspondence to: Geva Greenfield, Department of Primary Care and Public Health, School of Public Health, Imperial College London, 314 The Reynolds Building, St Dunstan's Road, London W6 8RP, g.greenfield@ic.ac.uk

For peer review only

For peer review only - http://bmjopen.bmj.com/site/about/guidelines.xhtml

Abstract

Objective: To summarise the literature on frequent attendances to hospital emergency departments and describe sociodemographic and clinical characteristics of children who attend EDs frequently.

Setting: Hospital emergency departments.

Participants: Children <21 years, attending hospital emergency departments frequently.

Primary outcome measures: Outcomes measures were defined separately in each study, and were predominantly the number of ED attendances per year.

Results: We included 21 studies representing 6,513,627 children. Between 0.3% to 75% of all paediatric ED users were frequent users. Most studies defined 4 or more visits per year as a "frequent ED" usage. Children who were frequent ED users were more likely to be less than 5 years old. In the US, patients with public insurance were more likely to be frequent attenders. Frequent ED users more likely to be frequent users of primary care and have long-term conditions; the most common diagnoses were infections and gastroenteritis.

Conclusions: The review included a wide range of information across various health systems, however children who were frequent ED users have some universal characteristics in common. Policies to reduce frequent attendance might usefully focus on preschool children and supporting primary care in responding to primary-care oriented conditions.

For peer review only - http://bmjopen.bmj.com/site/about/guidelines.xhtml

Article summary

Strengths and limitations of this study

- This is the first systematic review we are aware of, summarising the literature on frequent attendances of children to hospital emergency departments.
- Varied definitions for what constitute a frequent attendance introduces wide variations in the proportion of frequent attenders across the studies.
- Studies originating from different countries and healthcare systems makes comparisons between them challenging.

For peer review only

For peer review only - http://bmjopen.bmj.com/site/about/guidelines.xhtml

Funding/Support: This report is independent research supported by the National Institute for Health Research Applied Research Collaboration Northwest London. The views expressed in this publication are those of the authors and not necessarily those of the National Institute for Health Research or the Department of Health and Social Care. Grant No. NIHR200180. HQY was supported by the WHO Collaborating Centre for Public Health Education and Training at Imperial College London.

Ethical approval

No ethical approval was required for this article.

Competing interests statement. None of the authors have Conflict of Interest Disclosures.

Contributorship statement

Dr Greenfield, Ms Okoli and Dr Hayhoe were involved with conception and design, conducted the data analysis, and drafted the manuscript.

Ms Quezada Yamamoto was involved in conducting the literature searches, screening, data extraction and synthesis, and revised various versions of the manuscript.

Prof Blair, Prof Saxena and Prof Majeed were involved with acquisition of funding, conception and design, interpretation of the findings, provided clinical perspective, and revised various versions of the manuscript.

Data sharing statement

All data are available and could be shared upon request to the corresponding author.

Word count: 3,140 words, with one figure.

For peer review only

Introduction

Demand for health and emergency services is increasing rapidly driven by a worldwide epidemiological transition with increases in life expectancy, multi-morbidity, and burden from long-term conditions. For example, in England, the number of hospital emergency department (ED) attendances increased by 22% between 2008/09 to 2017/18 [1], while the growth in NHS England's funding have slowed significantly since 2010 [2], and approximately half of the NHS budget is currently spent on emergency and acute care, which is unsustainable in the long term [3], due to growing mismatch between demand and supply. These challenges have been intensified as health services are under increasing clinical and financial pressure amidst the COVID-19 pandemic.

Pressures on emergency health services are contributed to by a small group of individuals who attend EDs frequently, often referred to in the literature as 'Frequent attenders' [4,5]. For example, in Singapore, , the total visits made by frequent users cost four times as much as for non-frequent users, representing a significant economic burden [6]. For example, in England, young children, along with the elderly, are the group with the highest number of frequent attendances [7]. Children under 5 years old, while being 5.7% of the population [8], account for 25.1% of all frequent attenders [7]. Frequent attendance behaviour could be influenced by the emergency care models in each country, as well as the general healthcare system model and cultural differences between countries, however there are some commodities among frequent attending patients identified in the literature.

Approximately 15% of children ED attendances have been deemed inappropriate [9]. Nevertheless, the challenge with frequent ED visits is not only the over-utilisation of limited resources, but the potential harm that frequent exposure to ED can cause in terms of anxiety and distress [10].

For peer review only - http://bmjopen.bmj.com/site/about/guidelines.xhtml

Whilst frequent adult ED use has been reported in several reviews [11,12], there are currently two reviews of frequent children's ED attendances we are aware of. A review by Giannouchos et al. referred to American studies, and focused on mainstream conditions, excluding specific clinical subgroups of children [13]. Poku el al. focused on effectiveness of interventions for non-urgent cases [14]. This study aimed at reviewing the literature of paediatric frequent attenders in hospital EDs, focusing on the volume of frequent attendance and the demographic and clinical features of frequent attenders, with a view to better understanding how services might respond efficiently to parent and carer demand for ED services.

Methods

The review was performed according to the recommendation in the Cochrane Handbook for Systematic Reviews of Interventions [15]. The PRISMA guideline for the reporting of systematic reviews and meta-analyses [16]. was also followed.

Eligibility Criteria

Studies published in English from all over the world were included to ensure that results are generalizable. No publication year limit was placed on the search, to ensure inclusion of as many relevant studies as possible.

Participants

Given that there is no universally accepted threshold for defining frequent ED use, we included studies which referred to children who frequently use EDs. Studies of paediatric patients aged 21 and below presenting to the ED were included. The 21 years threshold was chosen to capture as many relevant studies as possible.

For peer review only

Outcome(s)

Studies had to have a measurable, definitive outcome of frequent attendances, for example a number of attendances per child per year

Study Design

Studies providing quantitative data on frequently attending children in ED were included. RCTs, controlled before and after (CBA) studies, and interrupted time series (ITS) studies were included. Cohort and cross-sectional studies related to frequent attendances of children were also included. Qualitative designs and case reports were excluded. Although observational studies are conducted in uncontrolled conditions, we included such studies in the searches as they can provide further evidence of the outcomes of frequent ED attendances of children

Information Sources

Medline, Embase, Maternity and Infant Care, PsycINFO, HMIC, Global Health and Scopus were searched using the Ovid interface.

Search Strategy

The search strategy was developed with the support of a librarian specialist. Search terms such as "emergency department", "accident adj2 emergency" were combined with search terms such as "adolescent* or child* or infant*" and "frequent admission/ visit" (Appendix 1). The search strategy was adapted for each electronic database and searches were run in June 2020.

Study Selection and Data Collection Process

The search results were transferred to Covidence (a systematic reviews management software) and further duplicates were removed. An initial screening of titles and abstracts was

For peer review only

For peer review only - http://bmjopen.bmj.com/site/about/guidelines.xhtml

followed by full-text screening of papers. The screening was done independently by OO, BH and GG. Discrepancies were resolved through discussion.

Data Extraction

The extracted information was collated into a structured format (Appendix 2). Additional information including sociodemographic information, reason(s) for visiting the ED, and relevant clinical information were extracted if were available.

Assessment of risk of bias in included studies

The risk of bias of included studies was assessed using the National Heart, lung and blood institute Quality Assessment Tool for Observational Cohort and Cross-sectional studies and Case Control studies [17]. It is an established and widely accepted Quality Assessment Tool. It was deemed appropriate because all included studies employed an observational study design. The criteria on the NIH Quality Assessment Tool are designed to help researchers focus on the key concepts for evaluating the internal validity of a study. They are not intended to create a list that could be tally up to arrive at a summary judgment of quality. The quality of the included studies was independently assessed by OO, BH and GG. Our responses to each of the 14 criteria are detailed in Appendix 3, followed by the criteria themselves. Discrepancies were resolved through discussion. Studies were not excluded based on risk of bias assessment.

Data Synthesis

Due to the heterogeneity in study designs and outcome measurements, a meta-analysis was deemed impractical. Instead a narrative synthesis approach was taken [18].

Patient and Public Involvement

Patients were not involved in the in the design of this study.

For peer review only

Results

Study Selection

Twenty-one studies representing 6,513,627 children met our inclusion criteria (Figure 1).

Study Characteristics

There were eighteen cohort studies [19–32] and three case control studies [33–35]. Fourteen were done in the USA, [21–26,28–31,35] two in Australia,[20,33] two in Canada,[27,36] one in the UK [32], one in Denmark [19] and one in Italy [34]. The study duration in most studies was one year. Frequently attending children percentage across all studies ranged from 0.3% to 75%. The data for all included studies were mainly extracted from ED registry, medical records and hospital admissions (Appendix 2).

Risk of bias within the included studies

Overall, the quality of all the included studies was high (Appendix 3). The main limitations related to risk of bias across the studies were sample size justification or power description, measurement of exposure prior to the outcome being measured, and assessment of the exposure more than once over time.

Results of individual studies

Definitions and rates of frequent attendances

Most studies used more than four or five ED visits in one year [23–25,32,34,37], although the definition varied remarkably. The proportion of frequent attendances ranges from 0.3% to 75% of the overall number of subjects. An Australian study [20] referred to children attending extensively, more than 20 times in a year. In a review of studies from the United States, frequently attending adult and children represented approximately 4.5% to 8% of all

For peer review only - http://bmjopen.bmj.com/site/about/guidelines.xhtml

ED users and 21% to 28% of all ED visits [12], which is a narrow range than the range reported here.

Characteristics of frequently attending children

The identified characteristics amongst frequently attending children were classified into 3 groups: demographic factors; health service factors and clinical characteristics.

(1) Demographic factors

Age and sex. Twelve out of twenty-one studies reported that children under the age of 5 were more likely to be FA compared to their older children [20–24,26,27,31–35], Infants who were frequent attenders have a higher ED visit rate in preschool years (subsequent 2 years) than those who were not FA in infancy [22]. Five out of the thirteen studies reporting gender differences found that boys were more likely than girls to attend frequently [20,21,25,32,34], whereas four indicated that girls were more likely to attend frequently [26,30,33,35], and one study reported no significance difference between boys and girls [21].

Ethnic Group. Findings regarding the association between ethnic groups and frequent attendances were mixed and specific to different cultures [21–24,28,30–32]. In six studies [21–23,25,30,38], children from black (Non-Hispanic) groups were more likely to attend EDs than children from white background. Another study reported that the proportion of White, Non-Hispanic (24.7% vs 24.1%) and Hispanic (55.0% vs 53.3%) were approximately equal [34]. In another study, children from Hispanic groups was the most frequent paediatric ED users [24]. One UK study reported that children from south Asian or Asian British groups attended most frequently relatively to other groups [32].

Income. Children from households with lower incomes were more likely to attend EDs frequently compared to children from households with either median or higher incomes

For peer review only

[27,28,30,32,39]. One study [24] found an association between median household income and paediatric frequent ED usage, and another reported that actually children living in cities with an average/high income were 1.2 times more likely to have frequent non-urgent ED attendances relatively to children living in low income cities [34].

Geographical residence and proximity to an ED. Frequently attending children were more likely to live closer to the ED when compared with non-frequent ED users [23,28,31,36,40]. Four studies reported that living within an urban location [23,24,30,34] was associated with a higher likelihood of being a frequent ED user. However, another study reported that living in a rural setting was associated with frequent ED use [28].

(2) health service and health insurance factors

Health Insurance. In nine out of the thirteen studies reported on the impact of health insurance of frequent attendances, frequently attending children were using public insurance [21,22,24–27,30–32], mainly Medicaid or State Assistance in the US. Patients with public insurance and uninsured patients had higher rates of frequent attendance compared with patients using private or commercial insurance [22,24,29,30,35].

Primary Care Provider. Among frequent ED users, the mean number of primary care visits per year was 6.2 visits [25]. Three studies indicated that frequent users of the ED were also more likely to be frequent users of primary care compared to non-frequent ED attenders [22–24]. One study found that frequently attending children who did not have a designated primary care physician were more likely to attend the ED more frequently compared to those who did have a primary care physician [22]. This was especially the case during the first three years of life. Another study reported frequent ED users were more likely to live in areas with many primary care providers [23].

For peer review only - http://bmjopen.bmj.com/site/about/guidelines.xhtml

Access to healthcare barriers. Barriers to access to healthcare services, such as difficulty in finding physicians and booking appointments alongside transportation challenges, played a significant role in frequent ED use. Caregivers who experienced cultural or language barriers were 5.8 times more likely to take their child to the ED more than twice within the period of 12 months compared to children who did not have caregivers who experienced cultural or language barriers [29]. Transport challenges were also correlated with numerous ED visits [22].

(3) Clinical factors

Clinical diagnoses. Having chronic conditions were associated with high frequency ED use [21,23,25,26,28,29,31–33]. The most common diagnoses among frequently attending children were upper respiratory tract infections[20,21,23,25–27,34], accounting for approximately one third of all frequent attendances. Other common diagnoses were viral infections [20,23,27] , gastroenteritis [21,23,25] and mental health problems [36]. Frequently attending children were also more likely to have a discharge diagnosis related to oncology, neurology, respiratory, endocrinology and psychiatric complaints, compared with occasional attenders who were more likely to present with injury or trauma [21,33]. However, a Hawaiian study recognised that patients with chronic conditions often presented to the ED with problems which were not related to their chronic condition [31]. An American study identified various conditions associated with frequent ED attendances in children, such as a history of increased tone (representative of neuromuscular disease), eczema, pneumonia, vocal cord dysfunction, technology dependence (gastrostomy tube or tracheostomy), and various allergies. Frequently attending children in this study had a higher likelihood of acquiring prescriptions for asthma medication or medication related to other diseases [30].

For peer review only - http://bmjopen.bmj.com/site/about/guidelines.xhtml

Physical injuries and maltreatment. Frequently attending children presented with superficial injuries such as dislocations, sprains, strain injuries, contusion, open wounds and other trauma to the skin [19,23]. Most of these incidents occurred in children aged 6-11 years old and adolescents [34]. Young children with higher frequency of ED visits are at significantly increased risk of being victims of child maltreatment compared to occasional users [35].

Arrival, admission and discharge. Frequently attending children were more likely to arrive to the ED by ambulance compared to occasional attenders [20,33]. They were more likely to have at least more than one ED visit leading to hospitalisation and/or transfer to another department, compared to non-frequent ED utilizers [21,24,33]. They were two times more likely to require admission for a mental health-related problem, compared to non-frequent ED utilizers [33]. They were 1.2 times more likely more likely to have received antibiotics and laboratory testing on discharge, compared to infrequent ED utilizers [24].

Discussion

Summary of main findings

The percentage of children who were frequent ED users ranged from 0.3% to 75%. Children who were frequent ED users were more likely to be aged less than 5 years, live in an urban location, near to an ED. Findings regarding sex, income and ethnicity were mixed. In the US, patients with public insurance were more likely frequent attendance compared with patients using private insurance. Frequent ED users more likely to be frequent users of primary care compared to non-frequent ED attenders. They tended to have long-term conditions and the most common diagnoses among frequently attending children were upper respiratory tract infections, viral infections and gastroenteritis.

For peer review only - http://bmjopen.bmj.com/site/about/guidelines.xhtml

Strengths and limitations

To our knowledge, this is the first systematic review to report on frequent ED attendance of children in a global perspective. Some challenges in the findings should be acknowledged. All the studies had observational design. The varied definitions for what constitute a frequent attendance, challenge the comparability of the studies, introducing wide variations in the proportion of frequent attenders between the studies. It is difficult to know whether the source of this variation is an outcome of differences in measurement methods, differences in populations, differences in healthcare systems structure or other sources. Likewise, the studies originated from different countries, cultures and healthcare systems, which makes comparisons between them challenging. Whereas most studies included all children <18 years, some studies focused on specific groups such as the <5 years old. Also, there was no blinding of the outcome assessor neither did majority of studies report whether there was a loss to follow-up. Finally, this systematic review restricted literature to only those that reported in English, which could potentially introduce selection bias.

Implications for future research

There is still little evidence on whether frequent ED users attend multiple ED sites or whether they are frequent users of other healthcare services. Linked data sets containing primary, secondary, hospital and social care data could be used for this purpose.

It would be useful to look at parent/carer characteristics as factors affecting frequently attending children. This is particularly important with younger children where the decision to attend the ED is made by the carer. This might highlight specific parent groups suspectable to frequent ED use.

From methodological perspective, using a uniform definition which will enable comparison across different studies. An alternative approach to the commonly used visit count approach

For peer review only

using a proportional threshold [41] (e.g., top 10% of all enlisted frequently attending children).

No data were found on any association between frequent ED attendances of children and risks of infection and other adverse health events following frequent ED visits. This is an important perspective to be looked at in future studies. Likewise, the fact that the studies come from high income countries might reflect a tendency for the frequent attendances behaviour to be more prominent matter in high-income countries, however it might also reflect little research interest in frequent attendance behaviour in LMIC. This might call for attention for frequent attendance in LMIC if challenges exist.

In relation to ethnicity, the findings were mixed. Ethnicity itself might manifest cultural differences and different attitudes toward the healthcare system and to medicine in general. While is it challenging to attribute clear characteristics to each ethnicity, the ethnicity still played a role in predicting frequent attendances. A more useful indicator was the association of frequent attendances with lower household income. A lower household income may imply stronger reliance on public insurance and public healthcare services, as was evident in the findings. Public healthcare services enable accessible healthcare to all or most people, however the lack of cost containment may contribute to frequent attendances. It is crucial though to establish the reasons for frequent attendances, whether they manifest from unresolved clinical matters, lack of self-care skills, mental health challenges or broader social determinants.

Implications for healthcare policy and practice

Creating tailored pathways to provide a response to the specific needs of frequently attending children are crucial. The characteristics of frequently attending children highlighted by this study can be used by healthcare providers to develop targeted interventions aimed at

For peer review only - http://bmjopen.bmj.com/site/about/guidelines.xhtml

providing tailored response to the needs of frequent ED users either through primary care or other healthcare services. EDs could highlight paediatric patients attending EDs frequently to their GP practice so their GP could identify and seek to address unmet healthcare needs.

Proactive identification of children who frequently attend EDs at the primary care end (through flagging ED discharge summaries, or through EDs highlighting frequent attendance to the GP), and reviewing their situation to identify possible areas of concern or failure in support, could potentially reduce reliance on ED. Some ED triage services in England already take this approach, where a triage nurse may admit the patient to the ED or may redirect the patient to their GP, to an urgent care centre, a wellbeing service or social care service.

Frequent attendances might be a feature of publicly funded systems where services are widely available to patients at no charge. Indeed, US data show that patients with public insurance and the uninsured had higher rates of frequent attendance compared with those using private or commercial insurance.

But rather than trying to contain frequent attenders using caps and charges, it might be useful to understand why frequent users keep coming to the ED, and whether frequent attendances stem from pure clinical matters, psycho-social factors, lack of health literacy or self-care skills, for example among first time parents, or other factors.

Access to primary care is cited as a main driver in ED visits in children, particularly during out of hours. [3,42]" While access to primary care cannot be directly implied as a reason for frequent attenders, it might be a potential explanation for frequent attendances. If parents face challenges in accessing primary care, particularly during out-of-hours, they will understandably seek hospital care. Ensuring that primary care is accessible and effective in meeting the needs of children and their carers will therefore be essential in reducing

For peer review only - http://bmjopen.bmj.com/site/about/guidelines.xhtml

unnecessary paediatric ED use. Children who use primary care benefit from health service accessibility, health prevention and early management of illnesses [43]. Improving access to primary care could hence reduce repeat ED use and improve overall long-term health.

Furthermore, as some clinical characteristics have been identified amongst frequently attending children, policy makers can implement national campaigns that will create awareness of what healthcare provider to visit with the appropriate associated symptoms.

Conclusion

The review included a wide range of information across various health systems, however children who were frequent ED users have some universal characteristics in common. We identified demographic factors, health service factors, and clinical characteristics relating to frequent ED attendances among children.

Policies to reduce frequent attendance might usefully focus on preschool children and supporting primary care in responding to primary-care oriented conditions. Further investigation of the potential of tailored pathways to respond to the specific health needs of frequently attending children will be valuable in identification of effective interventions to reduce frequent attendance and improve children's long-term health. Specifically, policies to reduce frequent attendance might usefully focus on preschool children and supporting primary care in responding to primary-care oriented conditions.

For peer review only - http://bmjopen.bmj.com/site/about/guidelines.xhtml

References

1 NHS England. A&E Attendances and Emergency Admissions. https://www.england.nhs.uk/statistics/statistical-work-areas/ae-waiting-times-and-activity/ (accessed 29 Nov 2017).

2 King's Fund. How hospital activity in the NHS in England has changed over time. https://www.kingsfund.org.uk/publications/hospital-activity-funding-changes (accessed 25 Jun 2018).

3 Cecil E, Bottle A, Cowling TE, *et al.* Primary Care Access, Emergency Department Visits, and Unplanned Short Hospitalizations in the UK. *Pediatrics* 2016;**137**:e20151492. doi:10.1542/peds.2015-1492

4 Greenfield G, Blair M, Aylin PP, *et al.* Frequent attendances at emergency departments in England. *Emerg Med J* 2020;**37**:597–9. doi:10.1136/emermed-2018-208189

5 Greenfield G, Blair M, Aylin PP, *et al.* Characteristics of frequent paediatric users of emergency departments in England: an observational study using routine national data. *Emerg Med J* 2021;**38**:146–50. doi:10.1136/emermed-2019-209122

6 Boh C, Li H, Finkelstein E, *et al.* Factors Contributing to Inappropriate Visits of Frequent Attenders and Their Economic Effects at an Emergency Department in Singapore. *Acad Emerg Med* 2015;**22**:1025–33. doi:10.1111/acem.12738

7 Greenfield G, Blair M, Aylin PP, *et al.* Frequent attendances at emergency departments in England. *Emerg Med J* 2020;:emermed-2018-208189. doi:10.1136/emermed-2018-208189

8 Principal projection - UK population in age groups - Office for National Statistics. https://www.ons.gov.uk/peoplepopulationandcommunity/populationandmigration/populationprojections/datasets/tablea21principalprojectionukpopulationinagegroups (accessed 16 Feb 2021).

9 McHale P, Wood S, Hughes K, *et al.* Who uses emergency departments inappropriately and when - a national cross-sectional study using a monitoring data system. *BMC Med* 2013;**11**:258. doi:10.1186/1741-7015-11-258

10 Nager AL, Mahrer NE, Gold JI. State Trait Anxiety in the Emergency Department: An Analysis of Anticipatory and Life Stressors. *Pediatr Emerg Care* 2010;**26**:897–901. doi:10.1097/PEC.0b013e3181fe90eb

11 Soril LJJ, Leggett LE, Lorenzetti DL, *et al.* Reducing Frequent Visits to the Emergency Department: A Systematic Review of Interventions. *PLOS ONE* 2015;**10**:e0123660. doi:10.1371/journal.pone.0123660

12 LaCalle E, Rabin E. Frequent Users of Emergency Departments: The Myths, the Data, and the Policy Implications. *Ann Emerg Med* 2010;**56**:42–8. doi:10.1016/j.annemergmed.2010.01.032

For peer review only

For peer review only - http://bmjopen.bmj.com/site/about/guidelines.xhtml

13 Giannouchos TV, Kum H, Foster MJ, *et al.* Characteristics and predictors of adult frequent emergency department users in the United States: A systematic literature review. *J Eval Clin Pract* 2019;**25**:420–33. doi:10.1111/jep.13137

14 Poku BA, Hemingway P. Reducing repeat paediatric emergency department attendance for non-urgent care: a systematic review of the effectiveness of interventions. *Emerg Med J* 2019;:emermed-2018-207536. doi:10.1136/emermed-2018-207536

15 Pollock M, Fernandes R, Becker L, *et al.* Cochrane Handbook for Systematic Reviews of Interventions. In: *The Cochrane Collaboration*. 2016. www.handbook.cochrane.org (accessed 16 Aug 2019).

16 Shamseer L, Moher D, Clarke M, *et al.* Preferred reporting items for systematic review and meta-analysis protocols (PRISMA-P) 2015: elaboration and explanation. *BMJ* 2015;**349**:g7647–g7647. doi:10.1136/bmj.g7647

17 Study Quality Assessment Tools | National Heart, Lung, and Blood Institute (NHLBI). https://www.nhlbi.nih.gov/health-topics/study-quality-assessment-tools (accessed 5 Aug 2019).

18 Rodgers M, Sowden A, Petticrew M, *et al.* Testing Methodological Guidance on the Conduct of Narrative Synthesis in Systematic Reviews: Effectiveness of Interventions to Promote Smoke Alarm Ownership and Function. *Evaluation* 2009;**15**:49–73. doi:10.1177/1356389008097871

19 Laursen B. Injury patterns in children with frequent emergency department visits. *Emerg Med J EMJ* 2006;**23**:59–60.

20 Gibson NP, Jelinek GA, Jiwa M, *et al.* Paediatric frequent attenders at emergency departments: A linked-data population study: Paediatric frequent attenders at emergency departments. *J Paediatr Child Health* 2010;**46**:723–8. doi:10.1111/j.1440-1754.2010.01829.x

21 Alpern ER, Clark AE, Alessandrini EA, *et al.* Recurrent and High-frequency Use of the Emergency Department by Pediatric Patients. *Acad Emerg Med* 2014;**21**:365–73. doi:10.1111/acem.12347

22 Cabey WV, MacNeill E, White LN, *et al.* Frequent Pediatric Emergency Department Use in Infancy and Early Childhood: *Pediatr Emerg Care* 2014;**30**:710–7. doi:10.1097/PEC.0000000000000233

23 Neuman MI, Alpern ER, Hall M, *et al.* Characteristics of Recurrent Utilization in Pediatric Emergency Departments. *PEDIATRICS* 2014;**134**:e1025–31. doi:10.1542/peds.2014-1362

24 Mueller EL, Hall M, Carroll AE, *et al.* Frequent Emergency Department Utilizers Among Children with Cancer. *Pediatr Blood Cancer* 2016;**63**:859–64. doi:10.1002/pbc.25929

For peer review only - http://bmjopen.bmj.com/site/about/guidelines.xhtml

25 Christensen EW, Kharbanda AB, Veiden HV, *et al.* Predicting Frequent Emergency Department Use by Pediatric Medicaid Patients. *Popul Health Manag* 2017;**20**:208–15. doi:10.1089/pop.2016.0051

26 Supat B, Brennan JJ, Vilke GM, *et al.* Characterizing pediatric high frequency users of California emergency departments. *Am J Emerg Med* Published Online First: 2018. doi:10.1016/j.ajem.2018.12.015

27 Seguin J, Osmanlliu E, Zhang X, *et al.* Frequent users of the pediatric emergency department. *CJEM* 2018;**20**:401–8. doi:10.1017/cem.2017.15

28 Zook HG, Kharbanda AB, Puumala SE, *et al.* Emergency Department Utilization by Native American Children: *Pediatr Emerg Care* 2018;**34**:802–9. doi:10.1097/PEC.0000000000001289

29 Taylor T, Salyakina D. Health Care Access Barriers Bring Children to Emergency Rooms More Frequently: A Representative Survey. *Popul Health Manag* 2019;**22**:262–71. doi:10.1089/pop.2018.0089

30 Das LT, Abramson EL, Stone AE, *et al.* Predicting frequent emergency department visits among children with asthma using EHR data. *Pediatr Pulmonol* 2017;**52**:880–90. doi:10.1002/ppul.23735

31 Yamamoto LG, Zimmerman KR, Butts RJ, *et al.* Characteristics of frequent pediatric emergency departments users. *Pediatr Emerg Care* 1995;**11**:340–6. doi:0749.5161/95/1106.0340/$03.33/0

32 Blair M, Poots A, Lim V, *et al.* Pre-school children who are frequent attenders in Emergency Departments: an observational study of associated demographics and clinical characteristics. *Arch Dis Child* 2017;**103**:19–23. doi:https://dx.doi.org/10.1136/archdischild-2016-311952

33 Markham D, Graudins A. Characteristics of paediatric frequent presenters to an Australian emergency medicine network: Paediatric presenters to emergency. *J Paediatr Child Health* 2013;**49**:950–4. doi:10.1111/jpc.12288

34 Riva B, Clavenna A, Cartabia M, *et al.* Emergency department use by paediatric patients in Lombardy Region, Italy: a population study. *BMJ Paediatr Open* 2018;**2**:e000247. doi:10.1136/bmjpo-2017-000247

35 Kuang X, Aratani Y, Li G. Association between emergency department utilization and the risk of child maltreatment in young children. *Inj Epidemiol* 2018;**5**:46. doi:10.1186/s40621-018-0176-5

36 Leon SL, Polihronis C, Cloutier P, *et al.* Family Factors and Repeat Pediatric Emergency Department Visits for Mental Health: A Retrospective Cohort Study. *J Can Acad Child Adolesc Psychiatry* 2019;**28**:9–20.

For peer review only

37 Vivier PM, Rogers ML, Gjelsvik A, *et al.* Frequent Emergency Department Use by Children. *Pediatr Emerg Care* Published Online First: 11 July 2019. doi:10.1097/PEC.0000000000001859

38 Hand BN, Boan AD, Bradley CC, *et al.* Emergency department utilization and monetary charges in adolescents with autism spectrum disorder, intellectual disability, and a population comparison group. *Autism Res* 2019;**12**:1129–38. doi:10.1002/aur.2124

39 Cabey WV, MacNeill E, White LN, *et al.* Frequent Pediatric Emergency Department Use in Infancy and Early Childhood: *Pediatr Emerg Care* 2014;**30**:710–7. doi:10.1097/PEC.0000000000000233

40 Portley R, North CS. Are There Differences in Pediatric Psychiatric Emergency Department Patients Related to Utilization Frequency? *Pediatr Emerg Care* 2020;**Publish Ahead of Print**. doi:10.1097/PEC.0000000000002010

41 Smits F, Brouwer H, ter Riet G. Persistent frequent attenders. *Br J Gen Pract* 2010;**60**:293.3-294. doi:10.3399/bjgp10X484002

42 Martin A, Martin C, Martin PB, *et al.* 'Inappropriate' attendance at an accident and emergency department by adults registered in local general practices: how is it related to their use of primary care? *J Health Serv Res Policy* 2002;**7**:160–5.

43 Starfield B, Shi L, Macinko J. Contribution of Primary Care to Health Systems and Health. *Milbank Q* 2005;**83**:457–502. doi:10.1111/j.1468-0009.2005.00409.x

For peer review only

Figures

Figure 1: PRISMA Flowchart

For peer review only

For peer review only - http://bmjopen.bmj.com/site/about/guidelines.xhtml

For peer review only

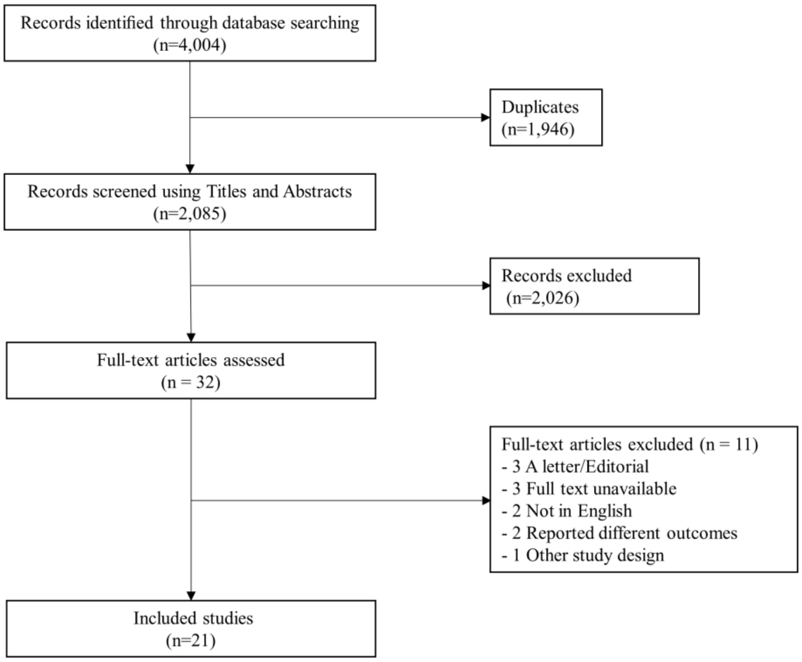

190x275mm (300 x 300 DPI)

Appendices

Appendix 1. Search terms

Search terms used in the Medline database.

1. exp Emergency Service, Hospital/
2. (Emergency adj3 (room* or department* or unit* or ward*)
3. (Trauma adj3 (room* or department* or unit* or ward*)
4. accident adj2 emergency
5. 1 or 2 or 3 or 4
6. adolescen* or child* or infant*
7. adolescent/ or exp child/ or exp infant/
8. p?ediatric*
9. baby or babies or teen* or girl* or boy*1
10. Youth*
11. young person*
12. young people
13. minor*
14. 6 or 7 or 8 or 9 or 10 or 11 or 13
15. frequen* adj3 (user* or attend* or usage* or admission* or visit*)
16. repeat* adj3 (user* or attend* or usage* or admission* or visit*)
17. heavy adj3 (user* or attend* or usage* or admission* or visit*)
18. Medical Overuse
19. over adj3 (user* or attend* or usage* or admission* or visit*)
20. super user*
21. superuser*
22. 15 or 16 or 17 or 18 or 19 or 20 or 21
23. 5 and 14 and 22
24. Limit 23 to English Language

For peer review only

Appendix 2. Study characteristics and main findings

**Author**	**Year**	**Study Design**	**Country**	**Data source**	**Definition of Frequent Attendance (Attendances per year)**	**Age**	**Duration of Study**	**Number (proportion) of frequently attending children**	**Main findings**
Yamamoto et al **[31]**	1995	Observational	USA	Kapiolani Medical Centre, Honolulu	>10	<21	4.6 years	72,049 (0.73%)	Frequent ED users had chronic disease conditions, neurological, gastrointestinal cardiac and endocrine conditions. Their Medical Insurance

For peer review only - http://bmjopen.bmj.com/site/about/guidelines.xhtml

									status included; private insurance, Medicaid/ state assistance, and no insurance
Laursen et al **[19]**	2006	Cohort	Denmark	National Patient Registry in Denmark	>5	<14	3 years	579,721 (0.43%)	Frequent ED users had more superficial injuries and more dislocations, sprains, and strains.
Gibson et al **[20]**	2010	Observational	Australia	Computerised Emergency Department Records, Perth Hospitals	>5	<15	6.5 years	229,883 (3%)	Factors associated with frequent attendances were; male, younger, self-referred, respiratory /infectious

For peer review only

									disorders, arrive by ambulance, higher admission rates & serious chronic illness
Markham et al **[33]**	2013	Case Control	Australia	Electronic Patient Records and Medical Records database, Monash Health	>8	<18	1 year	34,392 (0.38%)	Factors associated with frequent attendances were; female, arrive by ambulance, require admission for a mental health-related problem, discharge diagnosis related to oncology, neurology,

For peer review only

									respiratory, endocrinology and psychiatric complaints.
Alpern et al**[21]**	2014	Retrospective Cohort	USA	Paediatric Emergency Care Applied Research Network (PECARN)	>1	<17	1 year	695,188 (36%)	Asthma - most common ED diagnosis. Recurrent visits were associated with younger age, black or Hispanic race or ethnicity, and public health insurance
Cabey et al **[22]**	2014	Cross-sectional	USA	Patient Medical Records of	>5	<18	3 years	20,595 (28.5%)	Frequent PED use was associated with lack of primary care

				Carolinas Medical Center/Levin Children's Hospital					physician, nonprivate insurance, black race, Hispanic ethnicity, and residence in a poverty-associated zip code.
Neuman et al **[23]**	2014	Retrospective longitudinal	USA	Paediatric Health Information System, US	>1	<18	1 year	1,896,547 (75.19%)	Frequent user was had higher percentage of visits without medication administration, testing, or hospital admission & was usually <1 years old

For peer review only

Mueller et al **[24]**	2016	Retrospective Cohort	USA	Paediatric Health Information System (PHIS) Database, Colombia	>4	<19	1 year	17,943 (14.66%)	Frequent utilization was associated with age 1-19 years, Hispanic ethnicity, and urban residence. Few children with cancer received no medication, laboratory, or imaging during their ED visit.
Das et al **[30]**	2017	Retrospective Cohort	USA	Electronic Health Records (EHR) Systems,	>2	<18	1 year	2691(6.8%)	Frequent ED use was associated with co-morbidities, public insurance status, medication

				Weill Cornell Medical Center					history, and use of other healthcare resources. For publicly insured patients, the odds of frequent ED use were 3.1 times that of privately insured patients..
Christensen et al **[25]**	2017	Retrospective Cohort	USA	Medicaid	>4	<20	2 years	13,265 (8%)	Factors associated with frequent attendances were; chronic conditions, minority race, social determinants and young age.

Riva et al **[34]**	2018	Case-control	Italy	Administrative Healthcare databases of the Lombardy Region	>4	<18	1 year	1,640,713 (1.3%)	Trauma and respiratory tract infections were recognised as the most frequent diagnoses. Male gender, preschool age and place of residence were associated frequent ED use and frequent healthcare service use.
Supat et al **[26]**	2018	Retrospective Cohort	USA	California Office of Statewide	>6	<17	1 year	690,130 (2.3%)	Over 40% of frequent users visited 2 or more

				Health Planning and Development					hospitals. Frequent ED use were young age, publicly insured, and were admitted/transferred.
Blair et al **[32]**	2018	Observational	UK	District General Hospital Data, Brent and Harrow, London	>4	<5	1 years	10,169 (6.4%)	In the 10% sample, there were 304 attendances, and 69(23%) had an underlying chronic longstanding illness. This group were 2.4 times more likely to be admitted as in-patients.

Seguin et al **[27]**	2018	Retrospective Cohort	Canada	Systeme Informatique Urgence (SIURGE), electronic medical record database	>5	<18	1 year	52,088 (4.75%)	Lower socioeconomic status, Asthma, infectious ear, nose, and sinus disorders and other respiratory disorders were associated with a higher incidence of a recurrent visit.
Kuang et al **[35]**	2018	Nested Case Control	USA	California's Office of Statewide Health Planning	>2	<4	6 years	259,389 (1.45%)	For those with two or more ED visits Race/ethnicity, insurance status, and location of residence were also

For peer review

				(OSHPD) and Development					significantly associated with the risk of child maltreatment.
Zook et al **[28]**	2018	Cross-sectional	USA	Emergency Department Medical Records in Minnesota and South Dakota	>4	<18	1 years	112,746 (4.68)	Native American patients had a high proportion of mental health diagnoses. Frequent ED visitors had greater odds of NA race, age younger than 1-year, public insurance, female sex, residence within less than 5 miles from

For peer review only

									the ED, and chronic disease.
Taylor et al **[29]**	2019	Cross Sectional	USA	Telephone survey, South Florida	>2	<17	1 year	1057 (16%)	Multiple ED visits were most strongly associated with access to care barriers attributed to language and culture, trouble finding a doctor, scheduling an appointment, and transportation access.
Hand et al **[37]**	2019	Cross Sectional	USA	Administrative billing data for	>10	12-17	6 years	5001 (5%)	Majority of high ED utilizers were male,

				ED visits, South Carolina (SC) Revenue and Fiscal Affairs (RFA) Office					with the exception of the ASD-only group which was predominantly female. The most common insurance type among high ED utilizers was either Medicare or Medicaid.
Vivier et al **[36]**	2019	Retrospective Cohort	USA	Statewide hospital network, Rhode Island	>4	<17	1 year	172,844 (5.2%)	Frequent ED users were often between the age of 0-1, Hispanic, Spanish-speaking, publicly insured and lived in

For peer review

									areas with the highest neighbourhood risk. High ED utilizers were more associated with non-complex or complex chronic conditions and decreased distance to the ED.
Portley and North **[38]**	2020	Retrospective Cohort	USA	Children's Medical Center, The University of Texas Southwestern	>1	<18	1 year	6950 (10%)	Majority of frequent ED users were predominantly white English-speaking females who have private insurance

				Medical Center					and lived predominantly within the Dallas region of Texas, USA. Behavioural problems (34%) were the most common reason listed for the visit followed by suicidal ideation (24%) and suicide attempt (10%).
Leon et al **[39]**	2018	Retrospective Cohort	Canada	Children's Hospital of	>1	6-18	1 year	266 (26.3%)	Repeat ED visitors were commonly female who loved

For peer review only

				Eastern Ontario					within close proximity to the ED. Having a parent with a history for treatment of mental health (MH) was significantly associated with more frequent repeat visits to the ED. Furthermore, having more severe MH symptoms was associated with more frequent visits to the ED.

For peer review only

Appendix 3. Quality Assessment (Risk of Bias)

Quality Assessment of the included studies in the Systematic Review

Author	Study Design	Q1	Q2	Q3	Q4	Q5	Q6	Q7	Q8	Q9	Q10	Q11	Q12	Q13	Q14
Yamamoto et al [31]	Observational	Y	Y	Y	Y	N	Y	Y	Y	N/A	N	Y	N/A	N	N/A
Laursen et al [19]	Cohort	Y	Y	Y	Y	N	N	Y	N/A	N	N	Y	N	Y	Y
Gibson et al [20]	Observational	Y	Y	Y	Y	N	N	Y	N/A	Y	N	Y	N	N/A	Y
Alpern et al[21]	Retrospective Cohort	Y	Y	Y	Y	Y	Y	Y	N/A	Y	N	Y	N/A	Y	Y
Cabey et al [22]	Cross-sectional	Y	Y	Y	Y	N	N	Y	Y	Y	N	Y	N/A	Y	Y
Neuman et al [23]	Retrospective longitudinal	Y	Y	Y	Y	N	N	Y	Y	Y	N	Y	N/A	Y	Y
Mueller et al [24]	Retrospective Cohort	Y	Y	Y	Y	N	Y	Y	N	N/A	N	Y	N/A	N/A	Y
Das et al [30]	Retrospective Cohort	Y	Y	Y	Y	N	N	Y	N	N/A	N	Y	N/A	N/A	Y
Christensen et al [25]	Retrospective Cohort	Y	Y	Y	Y	N	Y	Y	N	N/A	N	Y	N/A	N	Y

Supat et al [26]	Retrospective cohort	Y	Y	Y	Y	N	Y	Y	N	Y	N	Y	N/A	N/A	Y
Blair et al [32]	Observational	Y	Y	Y	Y	N	Y	N	Y	N/A	N	Y	N/A	N/A	Y
Seguin et al [27]	Retrospective Cohort	Y	Y	Y	Y	N	N	Y	N	N/A	N	Y	N/A	Y	Y
Zook et al [28]	Cross-sectional, Cohort	Y	Y	Y	Y	N	N	Y	N	N/A	N	Y	N/A	N/A	Y
Leon et al [39]	Retrospective Cohort	Y	Y	Y	Y	N	Y	Y	N	N/A	N	Y	N/A	N/A	Y
Portley and North [38]	Retrospective Cohort	Y	Y	Y	Y	N	N	Y	N	N/A	N	Y	N/A	N/A	Y
Vivier et al [36]	Retrospective Cohort	Y	Y	Y	Y	N	Y	Y	N	N/A	N	Y	N/A	N/A	Y
Hand et al [37]	Cross Sectional	Y	Y	Y	Y	N	N	Y	N	N/A	N	Y	N/A	N/A	Y
Taylor et al [29]	Cross Sectional	Y	Y	Y	Y	N	Y	Y	N/A	N/A	N	Y	N/A	N	Y
Markham et al [33]	Retrospective case control	Y	Y	N	Y	Y	Y	Y	N	N	Y	N	Y		
Riva et al [34]	Case-control	Y	Y	N	Y	Y	N/A	Y	N	N	N	N	Y		

Kuang et al [35]	Nested Case Control	Y	Y	Y	N	Y	N/A	N/A	Y	Y	Y	Y	Y		

Legend:

Y Yes, N No, N/A (not applicable or not available)

For peer review only

Questions for the Tool for Quality Assessment of Observational Cohort and Cross-Sectional Studies [17]

1. Was the research question or objective in this paper clearly stated?
2. Was the study population clearly specified and defined?
3. Was the participation rate of eligible persons at least 50%?
4. Were all the subjects selected or recruited from the same or similar populations (including the same time period)? Were inclusion and exclusion criteria for being in the study prespecified and applied uniformly to all participants?
5. Was a sample size justification, power description, or variance and effect estimates provided?
6. For the analyses in this paper, were the exposure(s) of interest measured prior to the outcome(s) being measured?
7. Was the timeframe sufficient so that one could reasonably expect to see an association between exposure and outcome if it existed?
8. For exposures that can vary in amount or level, did the study examine different levels of the exposure as related to the outcome (e.g., categories of exposure, or exposure measured as continuous variable)?
9. Were the exposure measures (independent variables) clearly defined, valid, reliable, and implemented consistently across all study participants?
10. Was the exposure(s) assessed more than once over time?
11. Were the outcome measures (dependent variables) clearly defined, valid, reliable, and implemented consistently across all study participants?

For peer review only

12. Were the outcome assessors blinded to the exposure status of participants?

13. Was loss to follow-up after baseline 20% or less?

14. Were key potential confounding variables measured and adjusted statistically for their impact on the relationship between exposure(s) and outcome(s)?

Questions for the Tool for Quality Assessment of Case-Control Studies [17]

1. Was the research question or objective in this paper clearly stated and appropriate?
2. Was the study population clearly specified and defined?
3. Did the authors include a sample size justification?
4. Were controls selected or recruited from the same or similar population that gave rise to the cases (including the same timeframe)?
5. Were the definitions, inclusion and exclusion criteria, algorithms or processes used to identify or select cases and controls valid, reliable, and implemented consistently across all study participants?
6. Were the cases clearly defined and differentiated from controls?
7. If less than 100 percent of eligible cases and/or controls were selected for the study, were the cases and/or controls randomly selected from those eligible?
8. Was there use of concurrent controls?

For peer review only

9. Were the investigators able to confirm that the exposure/risk occurred prior to the development of the condition or event that defined a participant as a case?
10. Were the measures of exposure/risk clearly defined, valid, reliable, and implemented consistently (including the same time period) across all study participants?
11. Were the assessors of exposure/risk blinded to the case or control status of participants?
12. Were key potential confounding variables measured and adjusted statistically in the analyses? If matching was used, did the investigators account for matching during study analysis?

For peer review only
